# Reproductive life-history strategies in a species-rich assemblage of Amazonian electric fishes

**DOI:** 10.1371/journal.pone.0226095

**Published:** 2019-12-05

**Authors:** Joseph C. Waddell, Steve M. Njeru, Yasmine M. Akhiyat, Benjamin I. Schachner, Ericka V. Correa-Roldán, William G. R. Crampton

**Affiliations:** 1 Department of Biology, University of Central Florida, Orlando, Florida, United States of America; 2 Museo de Historia Natural, Universidad Nacional Mayor de San Marcos, Lima, Peru; Pontificia Universidade Catolica do Rio Grande do Sul, BRAZIL

## Abstract

The reproductive biology of only a small fraction of Neotropical freshwater fishes has been described, and detailed comparative studies of reproductive life-history variation in the Neotropical ichthyofauna are lacking. Here we describe interspecific variation in reproductive life history for a multi-species assemblage of the electric knifefish genus *Brachyhypopomus* (Hypopomidae: Gymnotiformes: Ostariophysi) from Amazonian floodplain and terra firme stream systems. During a year-round quantitative sampling program, we collected and measured key life-history traits from 3,410 individuals. Based on oocyte size distributions, and on circannual variation in gonadosomatic indices, hepatosomatic indices, and capture-per-unit-effort abundance of reproductive adults, we concluded that all species exhibit a single protracted annual breeding season during which females spawn fractionally. We found small clusters of post-larval individuals in one floodplain species and one terra firme stream species, but no signs of parental care. From analyses of body size-frequency distributions and otolith growth increments, we concluded that five species in our study area have approximately one-year (annual) semelparous life history with a single reproductive period followed by death, while two species have a two-year iteroparous life history, with breeding in both year-groups. Despite predictions from life-history theory we found no salient correlations between life history strategy (semelparity or iteroparity) and habitat occupancy (floodplain or terra firme stream). In the iteroparous species *B*. *beebei*, we documented evidence for reproductive restraint in the first breeding season relative to the second breeding season and argue that this is consistent with age-regulated terminal investment.

## Introduction

The Neotropical fish fauna is a promising system for exploring life-history evolution. Fishes in general are amenable to year-round quantitative sampling and exhibit suites of informative life history traits that can be measured from preserved specimens. Moreover, Neotropical fish assemblages are often represented by multiple closely-related species in adjacent habitats characterized by distinct structure, predator densities, nutrient availability, and other predictors of life history strategy [1, 2]. However, the reproductive ecologies of only a handful of the 6,000+ species of Neotropical freshwater fishes [3, 4] have been described in detail; see reviews by Loubens et al. [5], Lowe-McConnell [6], Menezes and Vazzoler [7], Ruffino and Isaac [8], Nuñez and Duponchelle [9]. Moreover, no studies have to date explored variation in life history across environmental gradients within a sympatric monophyletic group of closely related Neotropical fish species. Our understanding of comparative life history biology in the world’s most diverse aquatic vertebrate fauna is consequently limited [2, 10].

The Neotropical electric fish genus *Brachyhypopomus* (Hypopomidae, Gymnotiformes) is an attractive system for studying comparative reproductive biology. It comprises a monophyletic group of 28 species that occupy floodplains, swamps, river margins and small streams through most of lowland South America and Panama [11, 12]. Like other gymnotiforms, *Brachyhypopomus* generate a constant electric organ discharge (EODs) that allow them to be readily located with a portable electric fish finder and captured with almost 100% success. This unrivaled sampling efficiency makes the group ideal for quantitative (capture per unit effort-based) field surveys of reproductive status. Also, the reproductive biology of *Brachyhypopomus* is currently one of the best known among Neotropical fishes, with detailed autecological studies of three subtropical *Brachyhypopomus* species: *B*. *bombilla*, *B*. *draco*, and *B*. *gauderio*, from southern Brazil [13–18], and one tropical species from Panama, *B*. *occidentalis* [19]. Moreover, one representative of the genus, *B*. *gauderio*, is an established model system in neurobiology, neuroendocrinology, and ethology–providing a unique set of resources for understanding life history evolution; see for example reviews in Stoddard [20] Gavassa et al. [21] and Crampton [22]. Nonetheless, little is known about variation in the reproductive life history and ecology of *Brachyhypopomus* in the mega-diverse assemblages of Greater Amazonia (the Amazon, Orinoco, and Guianas), where the genus attains its highest diversity [11].

Here we present the results of a detailed survey of life history variation in a diverse, nine-species assemblage of *Brachyhypopomus* from the upper Amazon of Peru. Here the genus occupies two distinct habitats: first, whitewater floodplain lakes; second, low-order terra firme forest streams and adjacent shallow seasonal swamps. Amazonian floodplain lakes exhibit drastic seasonal fluctuation in water level [23], habitat availability [24], predator densities [25], and water chemistry [26] and are subject to greatly elevated low-water predation and mortality for small fishes [27]. Terra firme stream systems, which lie above the extent of seasonal river-floodplain inundation, are characterized by much lower annual variation in water level [28], habitat availability [29], community composition and abundance [30], and water chemistry [2, 31]. We conducted an intensive and uninterrupted year-long quantitative sampling program encompassing the entire annual river flood cycle and rainfall cycle, during which we sampled all *Brachyhypopomus* that we encountered with our electric fish finders. From the collected specimens we obtained a suite of measurements related to reproductive life history (including body size and mass, gonad mass, and liver mass) along with accompanying notes on distributions and seasonal habitat changes.

This contribution focuses on describing whether *Brachyhypopomus* species have either: (1) an approximately one-year (annual) life span with a single reproductive season followed by death (i.e. semelparity sensu Kirkendall & Stenseth [32], Roff [33], and Gavassa & Stoddard [34]; or (2) a multi-year life span with multiple reproductive seasons, i.e. iteroparity sensu Kirkendall & Stenseth [32] and Roff [33]. Previous field studies of two species of *Brachyhypopomus*, *B*. *gauderio* [16] and *B*. *occidentalis* [19] documented the disappearance of adult individuals following an annual breeding season, which is consistent with an annual, semelparous life history; see Gavassa et al. [21], Gavassa & Stoddard [34], and Sinnett & Markham [35].

Life history theory predicts that short-lived semelparous life histories should be favored under conditions of elevated predation [36], high productivity [37], and low ecosystem stability [38]; see reviews in Taylor et al. [39], Stearns [40], Roff [33]. Under these conditions, strategies for short lifespan and high intrinsic rates of population growth (i.e. r-selected strategies following the early heuristic of Pianka [41], or opportunist strategies in Winemiller’s [1] trilateral life-history model) are predicted to be favored over strategies for slower development, larger size and longer lifespan (K-selected strategies [41], or equilibrium strategists sensu Winemiller [1]); see Reznick et al. [42]. Floodplain systems typically exhibit higher levels of seasonal predation, higher ecosystem productivity, and less stability than terra firme stream systems [1, 27] and we therefore hypothesized that *Brachyhypopomus* species with annual, semelparous life histories should be more common than species with iteroparous life histories in floodplain systems, while iteroparous species should be more common in terra firme stream systems.

We also used our descriptions of life history in *Brachyhypopomus* to explore evidence for patterns of age-dependent reproductive terminal investment sensu Williams [43], whereby resources are diverted from growth and somatic maintenance to current reproduction as an animal’s residual reproductive value (i.e. its prospects for future reproduction) declines in later life stages [33, 40, 44]. Our analyses revealed an iteroparous life history and two-year lifespan in one abundant species, *B*. *beebei*. Therefore we predicted that this species should exhibit relative reproductive restraint in its first breeding season, with reproductive effort sustained solely by feeding (i.e. an ‘income breeding’ strategy sensu Jönsson [45]), but then switch to a ‘capital breeding’ strategy in its second and terminal breeding season, during which reproductive effort is sustained by the depletion of lipid energy stores.

Finally, in this contribution we also describe variation in other basic but poorly-known aspects of ecology and reproductive natural history, including spawning modality, reproductive movements, breeding seasonality, and the occurrence of parental care.

## Materials and methods

### The study system

**Fig 1:**
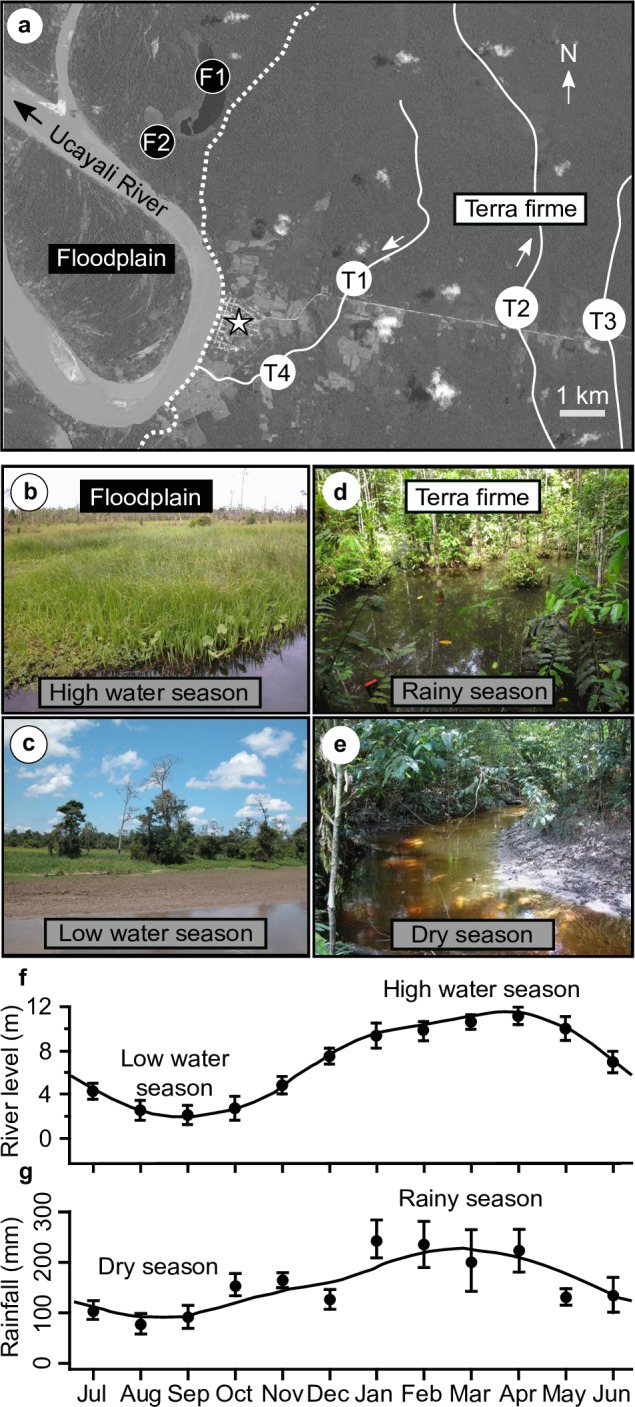
The study area and sampled habitats. a. Satellite image of vicinity of Jenaro Herrera (star), with floodplain lake sampling sites F1-F2 and terra firme stream sites T1-T4. Dotted line indicates low-water boundary of floodplain (to west) and terra firme systems. Arrows represent river/stream flow. b. Floodplain lake and macrophyte stand at high water. c. Floodplain lake at low water. d. Shallow swamp adjacent to terra firme stream in rainy season. e. Terra firme stream confined to its main course in dry season. f. Mean monthly Ucayali River level and g. mean monthly rainfall for 5-year period (2009–2013) from nearby (25 km) Requena meteorological station (data from Servicio Nacional de Meteorología y Hidrología del Perú); error bars represent one standard deviation.

Sampling was conducted near Jenaro Herrera in the upper Amazon basin of Peru (04°54' S, 073°39' W, Fig 1A) through a complete year beginning 1 March 2013 (S1 Appendix). We sampled two floodplain lake and four terra firme stream sites located in intact primary forest.

#### Floodplain systems

We sampled macrophyte stands in two lakes of the Holocene Ucayali River floodplain (sites F1–F2, Fig 1A); these were the only lakes within reach of our field base that retained water throughout the year. Typical of Amazonian whitewater floodplain lakes [2], sites F1–F2 are subject to a high-amplitude (9–11 m) annual inundation cycle (with a high-water maximum in March-April and a low water minimum in August-October, Fig 1F), are relatively nutrient-rich (electrical conductivity ca. 30–222 μS·cm^-1^, mean = 99, standard deviation [SD] = 73, with higher values during the high-water period), and exhibited variable levels of dissolved oxygen (0.2–1.8 mg·L^-1^, mean = 0.5, SD = 0.4, with hypoxia [< 0.5 mg·L^-1^]) throughout the high-water season), relatively high temperatures (27.5–33.8°C, mean = 30.7, SD = 1.7), variable pH (4.8–8.3, mean = 6.1, SD = 0.8) (S2 Appendix), and negligible or very slow flow (0–0.04 ms^-1^) [46].

#### Terra firme systems

We sampled submerged substrate (leaf litter, root mats, organic debris) at four sites in terra firme streams draining Paleogene-Neogene terra firme formations (Sites T1–T4, Fig 1A). At these sites we sampled both the main stream courses (Fig 1E) and adjacent seasonally-inundated swamps (Fig 1D), which reached their greatest extent during an annual rainy season from approximately January to April (Fig 1G). Typical of lowland Amazonian terra firme forest streams [2], sites T1-T4 were oligotrophic (electrical conductivity ca. 5–22 μS·cm^-1^, mean = 11, SD = 2.9), and in comparison to floodplain sites had higher levels of dissolved oxygen (1.6–6.0 mg·L^-1^, mean = 3.4, SD = 1.3) (but with levels in the 0.1–2 mg·L^-1^ range in adjacent terra firme swamps), low temperatures (23.0–28.4°C, mean = 26.4, SD = 0.8), lower pH (4.2–7.5, mean = 5.2, SD = 0.6) (S2 Appendix), and negligible to moderate flow (0–0.8 ms^-1^, typically 0.1–0.4 ms^-1^ in mid channel) [46].

### Quantitative sampling

Sampling was conducted from one hour after sunset to as late as 02:00, during the period of nocturnal activity of *Brachyhypopomus*. We located and captured all electric fish, in the order they were encountered, using a custom-made electric fish finder [47] and dipnet (Expedition Regular Hex Trapnet, Duraframe Dipnet, Viola, WI, with 3 mm mesh and 30.5 cm bag depth). This allowed us to quantify abundances by timed capture per unit effort (CPUE) (individuals·h^-1^). We visited each site from 1–10 times per month for 12 months (usually 2–6 times per month), for a total of 937 hours (S1 Appendix). The CPUE results reported herein are averaged across all sites from the same habitat type. To minimize negative impacts associated with repeated sampling (e.g. disruption of breeding, reduction of CPUE density), each sampling visit occurred at randomly chosen and never repeat-sampled ca. 100 m sections of the lake margin/stream course for a given site. In terra firme habitats, we devoted equal sampling attention to the main stream channel and to adjacent swamp habitats, when available. Field work and specimen exports were authorized by Dirección Regional de la Producción Regional de Loreto and by Instituto de Investigaciones de la Amazonía Peruana (IIAP). Sampling was conducted in non-indigenous protected territory administered by IIAP (sites F1–F2, T1–T3) or in private land with owner permission (site T4). No protected species were sampled.

All sampled fish were euthanized in 600 mg·L^-1^ eugenol and fixed in 10% buffered formalin. All body length and mass measurements, and gonad and liver mass measurements reported herein were taken from specimens fixed for two to three weeks, prior to storage in EtOH. Body length was measured as the distance from the snout to the posterior end of the anal fin (LEA), and as total length from snout to the caudal filament terminus (TL). *Brachyhypopomus* smaller than < 30 mm LEA penetrated the net mesh to varying degrees and were therefore eliminated from the analyses of size-frequency distributions described herein. We identified all species following the keys and differential diagnoses of Crampton et al. [11]. Small individuals of *B*. *beebei* and *B*. *verdii* were discriminated by the number of precaudal vertebrate, counted from digital radiographs using a Kodak DXS 4000 Pro X-ray machine. The (formalin-fixed) body mass (BM), gonad mass (GM), and liver mass (LM) of all specimens were measured with a Sartorius 64-IS balance (accurate to 0.1 mg). Animal use was approved by University of Central Florida Institutional Animal Care and Use Committee (Protocols 11-39W and 14-22W).

### Gonad maturation staging

**Fig 2:**
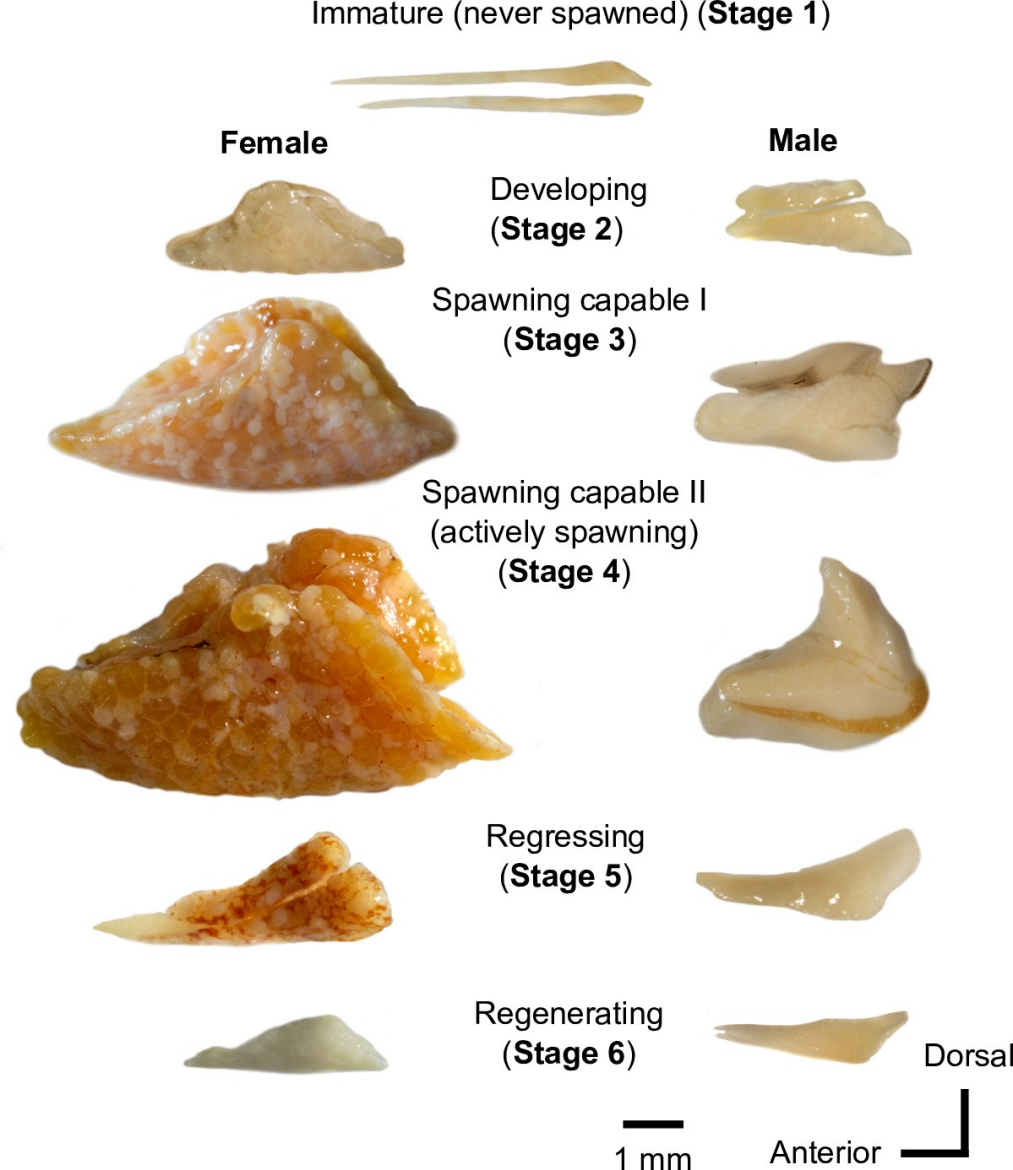
Gonads of *Brachyhypopomus* at the maturational stages described in Table 1. All images are from *B*. *beebei*, with the left side of each gonad pair in lateral aspect (only the left gonad is shown for male Stage 5). Black arrow indicates position where genital duct exits gonad and the point at which the gonads are severed in dissection. Gonads are scaled to the mean size for ca. 20 individuals of each sex and stage. Images were taken using the CamLift and Zerene software system described in Methods.

We determined the sex and maturational stage of all sampled *Brachyhypopomus* by visual assessment of macroscopic gonad morphology, using the six-stage scheme summarized in Table 1 and Fig 2. In brief: Stage 1 –Immature [never spawned]; Stage 2 –Developing; Stage 3 –Spawning capable (subphase I–not actively spawning); Stage 4 –Spawning capable (subphase II–actively spawning); Stage 5 –Regressing; Stage 6; Regenerating. This scheme is based on the standardized gonadal development scheme of Brown-Peterson et al. [48], and adapted from a scheme developed by Waddell & Crampton [49] for gymnotiforms in general (see S3 Appendix for equivalence of the scheme in Table 1 to that of Waddell & Crampton [49], and to the alternative universal scale of sexual maturation for oviparous teleost fish presented by Núñez and Duponchelle [9]). Waddell & Crampton [49] subjected a subset of gonads from each maturational stage to histological analysis to confirm that the stages based on gross anatomy corresponded to successive stages of oocyte and spermatozoan development from Brown-Peterson et al. [48].

**Table 1:** Gonadal maturation stages of *Brachyhypopomus* based on macroscopic analyses of gonadal morphology (see Fig 1).

Gonad stage (following Brown-Peterson et al. [47])	Gross anatomical features	Previous terminology listed by Brown-Peterson et al. [47] for equivalent stage
**Female**		
Stage 1: Immature (never spawned)	Ovaries thin, transparent, oocyte shapes not readily distinguishable. Gonads typically surrounded by mass of translucent polygonal/spherical lipocytes. Sex determination requires histological analysis.	Immature: Immature, virgin.
Stage 2: Developing	Ovaries with small, near-translucent or opaque white previtellogenic oocytes of approximately uniform size.	Maturing, early developing, early maturation, mid-maturation, ripening, previtellogenic.
Stage 3: Spawning capable (subphase I—not actively spawning)	Ovaries enlarged, with white to very pale yellow previtellogenic and vitellogenic oocytes. Individual may be approaching pre-spawning status for the first time, or in-between fractional spawning events.	Mature, late developing, late maturation, late ripening, total maturation, gravid, vitellogenic, ripe, partially spent, fully developed, prespawning, running ripe, final oocyte maturation, spawning, gravid, ovulated.
Stage 4: Spawning capable (Subphase II—actively spawning)	Ovaries swollen, with multiple size classes of yellow to orange, mostly vitellogenic oocytes. Oocytes may be released if abdomen of live specimen depressed.	
Stage 5: Regressing	Ovaries flaccid with stringy appearance, blood vessels prominent, usually with streaks of red-orange hemorrhaging; often a few remaining oocytes of disparate sizes scattered throughout.	Spent, regressing, postspawning, recovering.
Stage 6: Regenerating	For species with multi-year life histories. Ovaries thin, transparent, blood vessels present but reduced relative to Stage 5 (Regressing). Ovaries resemble those of immature (never spawned), but ovary wall thicker, and individuals belong to a larger size class.	Resting, regressed, recovering, inactive.
**Male**		
Stage 1: Immature (never spawned)	Testes thin and transparent; usually surrounded by mass of translucent, polygonal/spherical lipocytes. Sex determination requires histological analysis.	Immature, virgin.
Stage 2: Developing	Testes thin, near-translucent to whitish in color.	Maturing, early developing, early maturation, ripening.
Stage 3: Spawning capable (subphase I—not actively spawning)	Testes enlarged and thickened, whitish in color.	Late developing, mid-maturation, late maturation, late ripening, ripe, partially spent, running ripe, spawning.
Stage 4: Spawning capable (Subphase II—actively spawning)	Testes resembling stage 3 but more swollen with flattened lobular surfaces, white to pale cream in color. Milt often released with gentle pressure to abdomen.	
Stage 5: Regressing	Testes relatively thin and flaccid. Difficult to distinguish macroscopically from the developing stage.	Spent, regression, postspawning, recovering.
Stage 6: Regenerating	Testes relatively thin and transparent, difficult to distinguish macroscopically from immature (never spawned), but individuals belong to larger size class.	Resting, regressed, recovering, inactive.

Because our gonad-staging scheme is based on macroscopic assessments of gonadal morphology, it has two limitations. First, it cannot unambiguously determine the sex of all immature (never spawned) individuals (Stage 1) and regenerating individuals (i.e. representatives of species with multi-year lifespans that are undergoing gonadal regeneration prior to a second breeding season, Stage 6). Second, it is unable to discriminate unambiguously between developing (Stage 2) and regressing males (Stage 5) males. To account for these limitations, we combined all Stage 1 and 6 individuals into a single category in our analyses, and we also combined Stage 2 and Stage 5 males. We stress that the analyses of reproductive life history presented herein are unaffected by pooling these gonadal stages. Moreover, by basing our gonadal development scheme on simple macroscopic analyses alone, we were able to assign all 3,400+ individuals in this study to gonadal stage; a number for which individual histological analyses would have been intractable.

### Seasonal variation in reproductive status

For each month of the study period, we summed the CPUE abundance of all spawning capable (Stage 3 and 4) individuals of a given species and sex (averaged across all sites in the habitat in which the species occurs). Spawning capable fish are those which are developmentally and physiologically able to spawn [48]. Females in the active spawning subphase (Stage 4) are those in the process of releasing a fully-developed batch of oocytes, while males in the active spawning subphase are those in the process of releasing spermatozoa [48]. Females of fractionally spawning species (including all species of *Brachyhypopomus* in our study system, see Results ‘Oocyte size distributions and counts’) therefore effectively cycle back and forth between Stage 3 and 4 as successive batches are released in fractional spawning events.

For all sampled individuals we also calculated the GSI as (gonad mass/body mass)·100, and the hepatosomatic index (HSI) as (liver mass/body mass)·100. We calculated monthly averages of CPUE, GSI and HSI pooled for all individuals of a given species in a given habitat along with error bars of one standard deviation corresponding to variation between capture sites. We excluded from our GSI and HSI analyses all individuals in which ≥ 10% of ‘intact TL’ was missing due to caudal filament loss, unless the individual had regained ≥ 90% of intact TL by regeneration; we estimated intact TL from the head length (measured following [11]) via standard major axis regression of TL versus head length for all intact conspecifics of the same sex.

### Analysis of size-frequency distributions

With the aim of discriminating between species with one-year versus multi-year life spans, we utilized a mixture model approach [50] to recognize modal distributions in size-frequency distributions indicative of age cohorts. Mixture models have become popular for discriminating distinct modal groups in biological size-frequency datasets, e.g. Laslett et al. [51] and Sethi et al. [52]. They determine the most likely number of modes in a length-frequency dataset (among many theoretical permutations of modal number) using model performance estimates and then use optimization algorithms to fit one or more Gaussian distribution curves to the data around these modes. We used the Fraley et al. [53] Finite mixture model (FMM) implementation of mixture models, in the ‘mclust’ package [54] for R [55]. FMM uses a Bayesian information criterion approach to estimate model performance for modal number and applies expectation-maximization procedures sensu Dempster et al. [56] to compute best-fitting Gaussian distributions around identified modes.

We used LEA as our metric for body size in the FMMs instead of TL because LEA is less sensitive to caudal filament damage/regeneration and because some *Brachyhypopomus* species exhibit sexual dimorphism in caudal filament length [11, 57]. Using a custom-written R script, we submitted a vector of LEA to mclust (for all individuals of a given species). We then extracted the following parameters from the resulting best-fitting Gaussian distribution curve/s: mean, standard deviation, and scaling factor (relative height). These parameters were then used to superimpose the curve/s onto a size-frequency histogram (with bin-intervals of 10 mm LEA). Overestimation by the mixture models occasionally yielded more than one Gaussian distribution at a very similar modal value. In these cases, we manually removed the suspected redundant distribution (the width of which did not match the surrounding histogram contours) following Laslett *et al*. [51]. We excluded damaged individuals from our size-frequency analyses following the same criteria described above (‘Seasonal variation in reproductive status’).

### Otolith analyses

We further discriminated between species with one-year and multi-year life spans by identifying concentric dark and light bands in sagittal otoliths indicative of annular growth increments, i.e. ‘annuli’ sensu Campana [58]. These dark bands typically correspond to a period of decreased growth rate while the light bands are associated with periods of faster growth [59, 60]. For example, dark bands in the otoliths of *Cichla* were inferred to represent a period of slowed growth associated with reproduction [61]. The dark bands are in these cases the result of a spatial concentration of daily or quasi-daily growth increments, which are generally not possible to count accurately in small fish. Nonetheless, we are aware that some studies have reported a contrary pattern in which the dark bands represent a period of *increased* growth, e.g. Duponchelle et al. [62] for *Pygocentrus nattereri*, and we were therefore careful to consider whether a similar arrangement might occur in *Brachyhypopomus*. Following Campana [58], Morison et al. [63], and Fowler [60], we performed otolith analyses only on Stage 4 individuals in the upper 15% of the LEA distribution in species with a putative one-year lifespan, and the upper 15% of the LEA distribution for individuals of each putative year group in species suspected to exhibit multi-year lifespans. For all species and putative year groups we also confined our analyses to individuals sampled in the last two months of the breeding season (see Results: ‘Seasonal variation in reproductive status’). We removed the right sagittal otolith via dissection of the otic capsule, following removal of the operculum. Each otolith was then washed in distilled water, air-dried, and glass-slide mounted in a thermoplastic mounting adhesive (Crystalbond 509, Aremco Inc., Valley Cottage, NY), with the transverse plane oriented parallel to the slide [60, 64]. The otolith was then filed down on one side with increasingly fine grades of wet sandpaper (from 25 to 8 μm), the plastic melted to allow the entire otolith to be inverted, and the filing processing repeated on the other side until a thin and translucent transverse section remained. Following Morison et al. [63] we counted concentric dark bands indicative of annular growth increments from these slide preparations under a back-lit stereomicroscope. Our counts were conducted independently by three experienced researchers (henceforth ‘counters’) at the Florida Fish and Wildlife Conservation Commission Age and Growth Laboratory (St. Petersburg, FL). We scored an otolith as possessing a given number of bands only if all three counters agreed on the same number, or if two agreed and one was undecided. We scored the otolith as uncountable if two or more counters were unable to reach a count due to poor preparation quality (on no occasion did the counters reach conflicting band counts for the same otolith preparation).

The digital images of representative slide-mounted otoliths reported herein were taken at multiple focal planes using a Canon EOS 7D camera and 65 mm macro lens (set to f/16) attached to a Visionary Digital P-51 CamLift system (Dun, Inc.) and merged using Zerene Stacker software (Zerene Systems LLC, Richland, WA).

Finally, we performed marginal increment analysis (MIA) to validate annual formation of opaque bands, i.e. true annular growth increments (annuli), in otoliths following Campana [58], Lessa et al. [65], Lin and Tzeng [66], and Perez and Fabre [67]. The ‘marginal increment ratio’ is a measure used to determine the timing of opaque band formation. When the ratio is at its maximum, the most recent opaque band is furthest from the edge of the otolith, and when the ratio is at its minimum, the most recent band is close to the edge (and thus recently formed). Therefore, if an opaque band is to be presumed a true annulus (resulting from a period of slower growth), the marginal increment ratio should be at a minimum only once per year (just after formation) and is expected to approximate a sinusoidal pattern through the year in individuals sampled from a single location [58, 65–67].

Studies of ageing in multiple closely-related fish species typically select one species for MIA to validate that the growth bands constitute true annuli [58, 60]. We performed MIA on the floodplain species *B*. *bennetti*, based on 35 randomly-selected specimens sampled around the year from site F1. For each otolith, the following point-to-point linear measurements were measured with the ocular micrometer of a Meiji Techno RZ stereomicroscope: the radius of the otolith (R_C_), the distance from the center of the otolith core to the center of the most recently formed annulus (R_L_), and the distance from the center of the otolith core to the center of the oldest (inner-most) annulus (if present) (R_L-1_) [65, 66].

### Oocyte size distributions and counts

We examined the size-frequency distributions of oocytes to determine whether *Brachyhypopomus* exhibit a pattern of fractional spawning (also known as batch spawning), in which a female releases multiple successive batches of eggs during a single reproductive period [68, 69]. Fractional spawning contrasts with ‘total spawning’–where a single batch of eggs is released during a single reproductive period. We randomly selected up to seven females per species (or putative year group) with Stage 4 ovaries, hydrated the 70% EtOH-stored ovary pair for 48 hours in 1% formalin, gently dislodged the oocytes with microdissection tools in a moistened petri dish, and measured the diameters of a random subsample of 200 oocytes taken from both ovary pairs to the nearest 0.01 mm, using a stereomicroscope ocular micrometer. Comparable studies have counted from 50–200 oocytes [70, 71], including in other gymnotiform fishes [13]. We then applied FMMs (as described above for body size-frequency distributions) to superimposed best-fitting Gaussian distribution curve/s onto size-frequency histograms of egg size, with bin-intervals of 0.1 mm oocyte diameter.

For each of the individuals subjected to oocyte size-frequency analysis we also counted the total number of oocytes in the ovary pair. We then estimated the total number of oocytes belonging to the largest oocyte size group (those above the size of the overlap between the Gaussian distributions for the largest size group and the next smallest group) by multiplying the proportion of the 200 randomly chosen oocytes used for size-frequency analysis by the total oocyte number.

### Parental care

Parental care has been documented from field studies of *Gymnotus* by the detection of aggregations of post-larval or small juvenile individuals around single adult males (‘nests’) with electric fish finders [72]. These nests generate a readily detectable pattern of multiple simultaneous pulses in the loudspeaker output of the fish finder. We searched for signs of similar parental care in *Brachyhypopomus* during our fish finder-aided quantitative sampling program.

## Results

### The *Brachyhypopomus* fauna

Excluding individuals < 30 mm LEA and individuals with disqualifying levels of caudal filament damage, we collected 3,410 *Brachyhypopomus*–four species from floodplain lakes: *B*. *bennetti* (n = 588), *B*. *flavipomus* (n = 45), *B*. *regani* (n = 2), *B*. *brevirostris* (n = 8), and five species from terra firme streams: *B*. *beebei* (n = 548), *B*. *benjamini* (n = 134), *B*. *sullivani* (n = 778), *B*. *verdii* (n = 756), and *B*. *walteri* (n = 539) (S2 Appendix). We also encountered *B*. *beebei* in floodplain lake sites (n = 11) and *B*. *brevirostris* in terra firme stream sites (n = 1) but excluded these from all our analyses because the sample sizes were small. Catalog numbers for voucher specimens are listed in Crampton et al. [11].

### Analysis of size-frequency distributions

**Fig 3:**
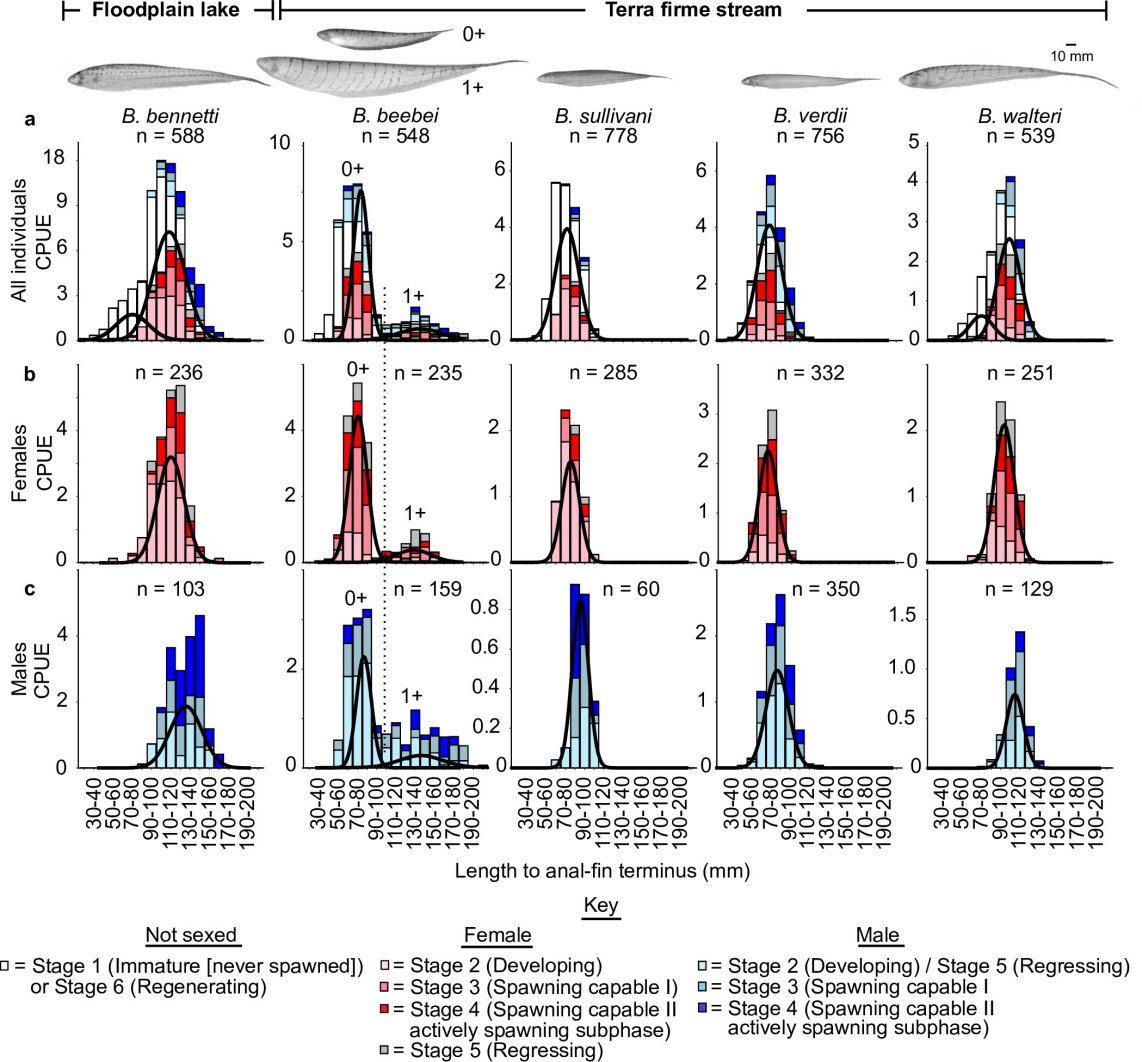
Size-frequency distributions for five species of *Brachyhypopomus* pooled over the 12-month study period with Gaussian distributions fitted by finite mixture models to show modal size groups. a. Unsexed immature/regressing individuals, females, and males combined (i.e. gonad maturation Stages 1–6). b. Females only (gonad maturation Stages 2–5). c. Males only (gonad Stages 2–5). CPUE = capture per unit effort in individuals·hr^-1^. Vertical dotted line shows that the cut-off between the putative 0+ and 1+ year groups of *B*. *beebei* at 115 mm LEA is the same for males and females. Stage 6 (regenerating) individuals are confined to the 1+ year group of *B*. *beebei*.

Based on size-frequency data pooled for all twelve months of the study (Fig 3), Gaussian functions fitted by FMMs revealed *unimodal* distribution of body sizes in females (Fig 3B) and males (Fig 3C) (at gonad maturation Stages 2–5) of four species: *B*. *bennetti*, *B*. *sullivani*, *B*. *verdii*, and *B*. *walteri*. In contrast, one species, *B*. *beebei*, exhibited a *bimodal* distribution of body size in both sexes, with the first group comprising individuals in the size range > 0 ≤ 115 mm LEA, and the second comprising individuals in the size range > 115 mm ≤ 210 mm LEA. We excluded *B*. *brevirostris*, *B*. *flavipomus*, *B*. *regani* from these analyses because of small sample sizes and we excluded *B*. *benjamini* because of poor sample representation during several months of the study. The size distributions reported in Fig 3B and 3C report sexual dimorphism of body size in all species, including in both size-groups of *B*. *beebei*. In all cases males exhibit a larger modal size than females. However, the modal groups corresponding to males and females were not distinguished by FMMs in which males, females and juveniles were combined (Fig 3A).

**Fig 4:**
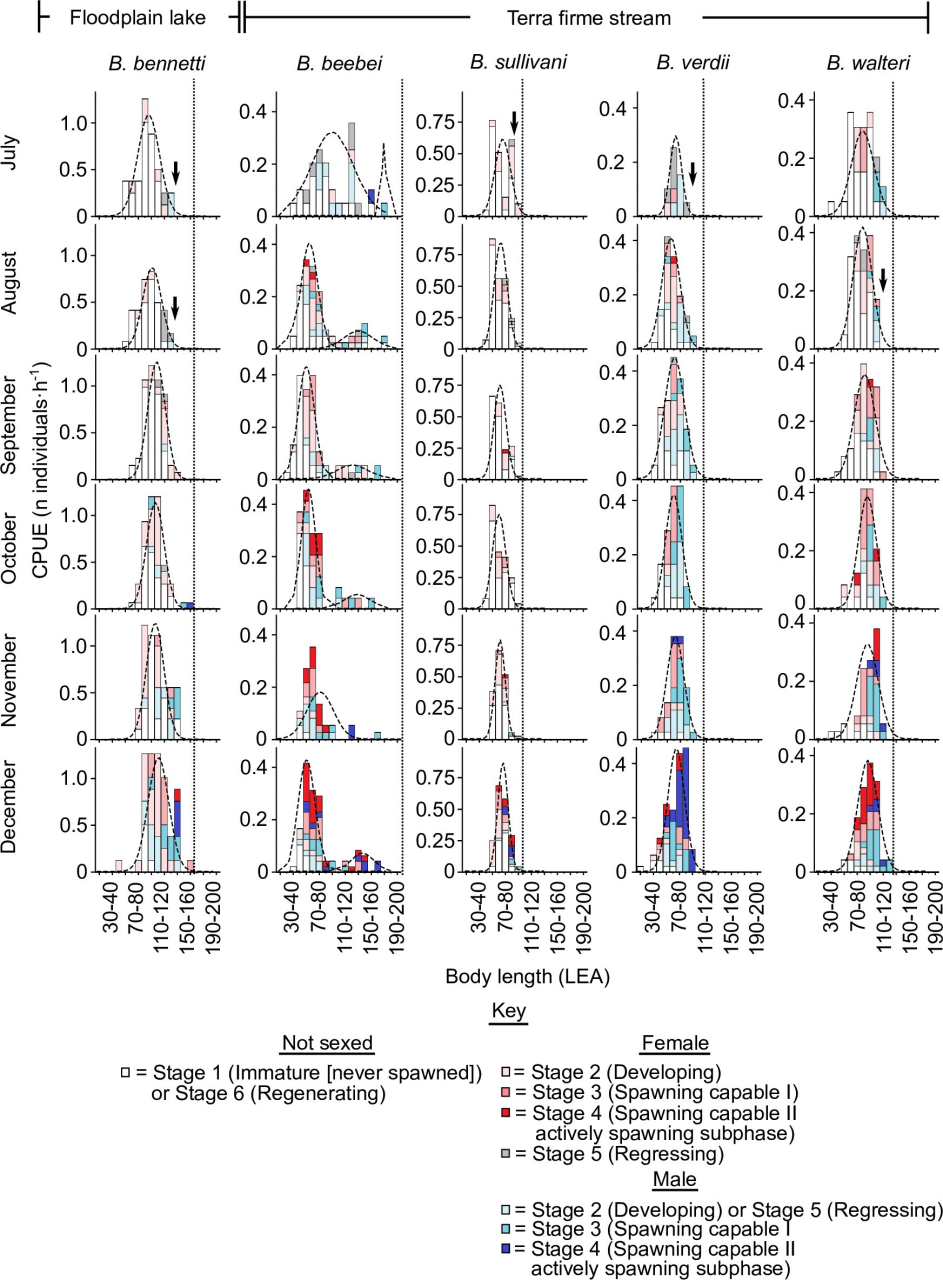
July–December monthly size-frequency distributions for five species of *Brachyhypopomus* with Gaussian distributions fitted by finite mixture models to show modal size groups. CPUE = capture per unit effort. Vertical dotted lines report maximum body size as length to anal-fin terminus (LEA). Vertical arrows indicate reduction/disappearance of large adult size classes in the late breeding season. Stage 6 (regenerating) individuals are confined to the 1+ year group of *B*. *beebei*.

**Fig 5:**
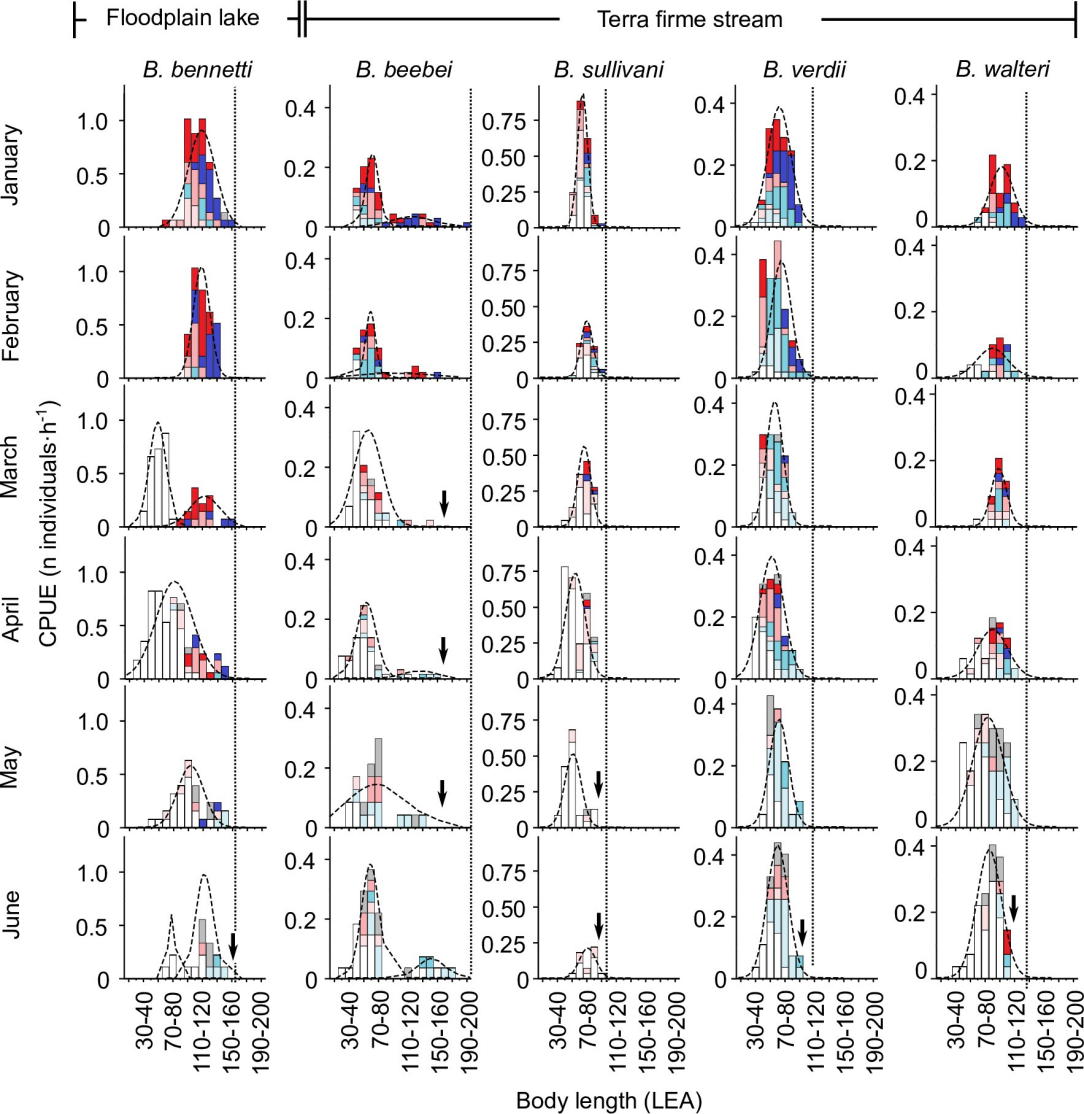
January–June monthly size-frequency distributions for five species of *Brachyhypopomus* with Gaussian distributions fitted by finite mixture models to show modal size groups. See Fig 5 caption and key for further details.

The raw monthly size-frequency data used to construct the pooled summary in Fig 3 are reported in Fig 4 (July to December) and Fig 5 (January to June). Here the FMMs again revealed unimodal size distributions for *B*. *bennetti*, *B*. *sullivani*, *B*. *verdii*, and *B*. *walteri*. In these four species we observed a bimodal distribution in only two months (March and June for *B*. *bennetti*, Fig 5) and in both these months the smaller group comprised *only* immature individuals (which we presume to be recently-recruited juveniles) while the larger group comprised mostly reproductively mature adults (gonad Stages 3–5). Likewise, a small group of immature individuals with a modal size of 70–80 mm LEA was recovered in *B*. *bennetti* and *B*. *walteri* in the pooled 12-month data for all individuals (Fig 3A), which we also presume correspond to cohorts of newly-recruited individuals. In contrast to the patterns observed in *B*. *bennetti*, *B*. *sullivani*, *B*. *verdii*, and *B*. *walteri*, the monthly size-distributions for *B*. *beebei* reported in Figs 4 and 5 exhibit two distinct size distributions (> 0 ≤ 115 mm LEA and > 115 mm ≤ 210 mm LEA) throughout the year, each of which comprise unsexed immature (Stage 1) or regenerating (Stage 6) individuals mixed with developing, spawning capable, and regressing adults (Stages 2–5).

Reproductive adults of *B*. *bennetti*, *B*. *sullivani*, *B*. *verdii*, and *B*. *walteri*, as well as adults belonging to the larger of the two size groups of *B*. *beebei*, disappeared from our samples near to and after the end of the annual breeding period (see ‘Seasonal variation in reproductive status’, below), typically during the period May–August (see vertical arrows superimposed onto monthly size distributions in Figs 4 and 5). This observation is consistent with post-reproductive mortality, which has been reported previously in field studies of two *Brachyhypopomus* species–*B*. *gauderio* from Uruguay [16], and *B*. *occidentalis* from Panama [19], and from aquarium studies of two species–*B*. *gauderio* [73] and *B*. *pinnicaudatus* [74].

### Otolith analyses

**Fig 6:**
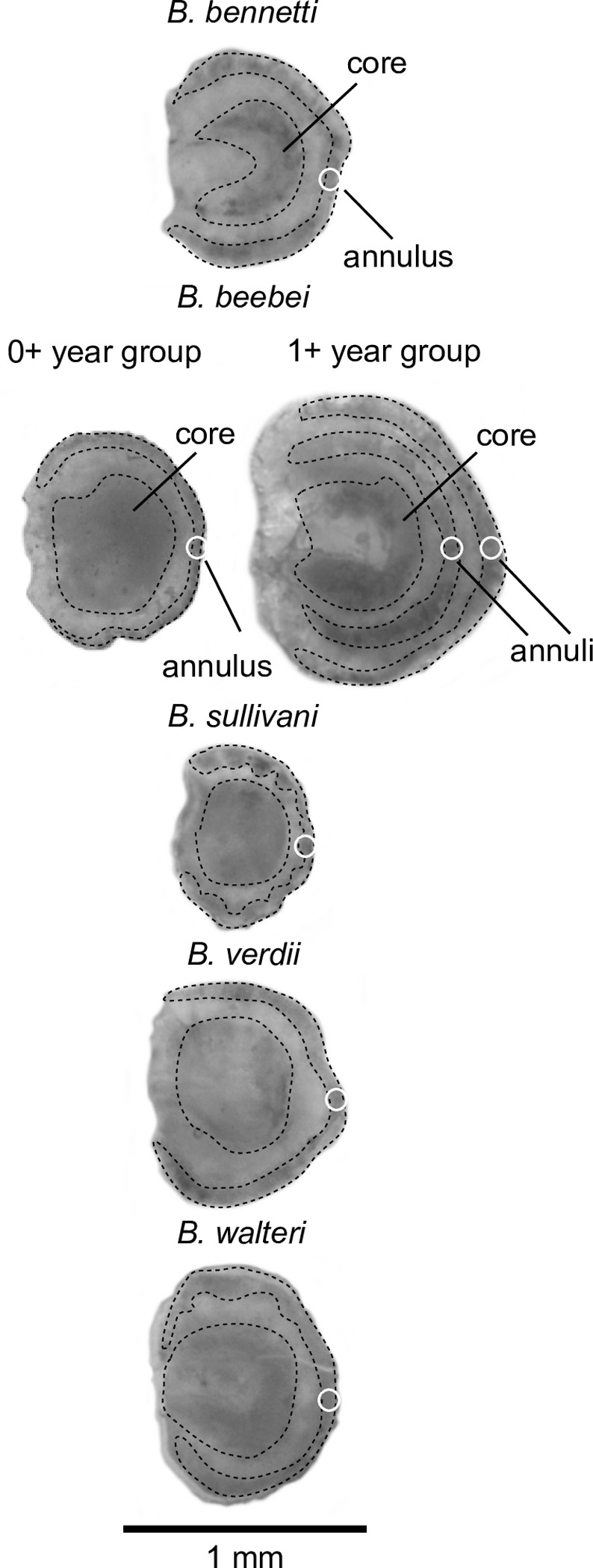
Sagittal otolith preparations for representative Stage 4 individuals of five species of *Brachyhypopomus*. Dotted lines are superimposed to highlight the positions of the otolith core and dark annuli. Note the single dark annulus in species with one-year semelparous (annual) lifespans: *B*. *bennetti*, *B*. *sullivani*, *B*. *verdii*, *B*. *walteri*. Note the single dark annulus in the 0+ year group of the iteroparous species *B*. *beebei*, but two dark annuli in the 1+ year group.

Large, Stage 4 individuals of four species presented a single dark annulus, near the edge of the otolith (Fig 6): *B*. *bennetti* (n = 5 of 5 examined preparations), *B*. *sullivani* (n = 4 of 5 preparations), *B*. *verdii* (n = 4 of 6 preparations), and *B*. *walteri* (n = 4 of 4 preparations) (see S4 Appendix for results of three independent counts, and specimen data).

In contrast, *B*. *beebei* presented a single dark annulus (also near the edge of the otolith) in large individuals of the smaller size group (> 0 ≤ 115 mm LEA) (n = 4 of 4 preparations), but two distinct dark annuli in the otoliths of large individuals of the larger size group (> 115 mm ≤ 210 mm LEA) (n = 4 of 4 preparations): an outer annulus at the otolith edge, and an inner one approximately midway between the core and the outer one (Fig 6) (S4 Appendix).

**Fig 7:**
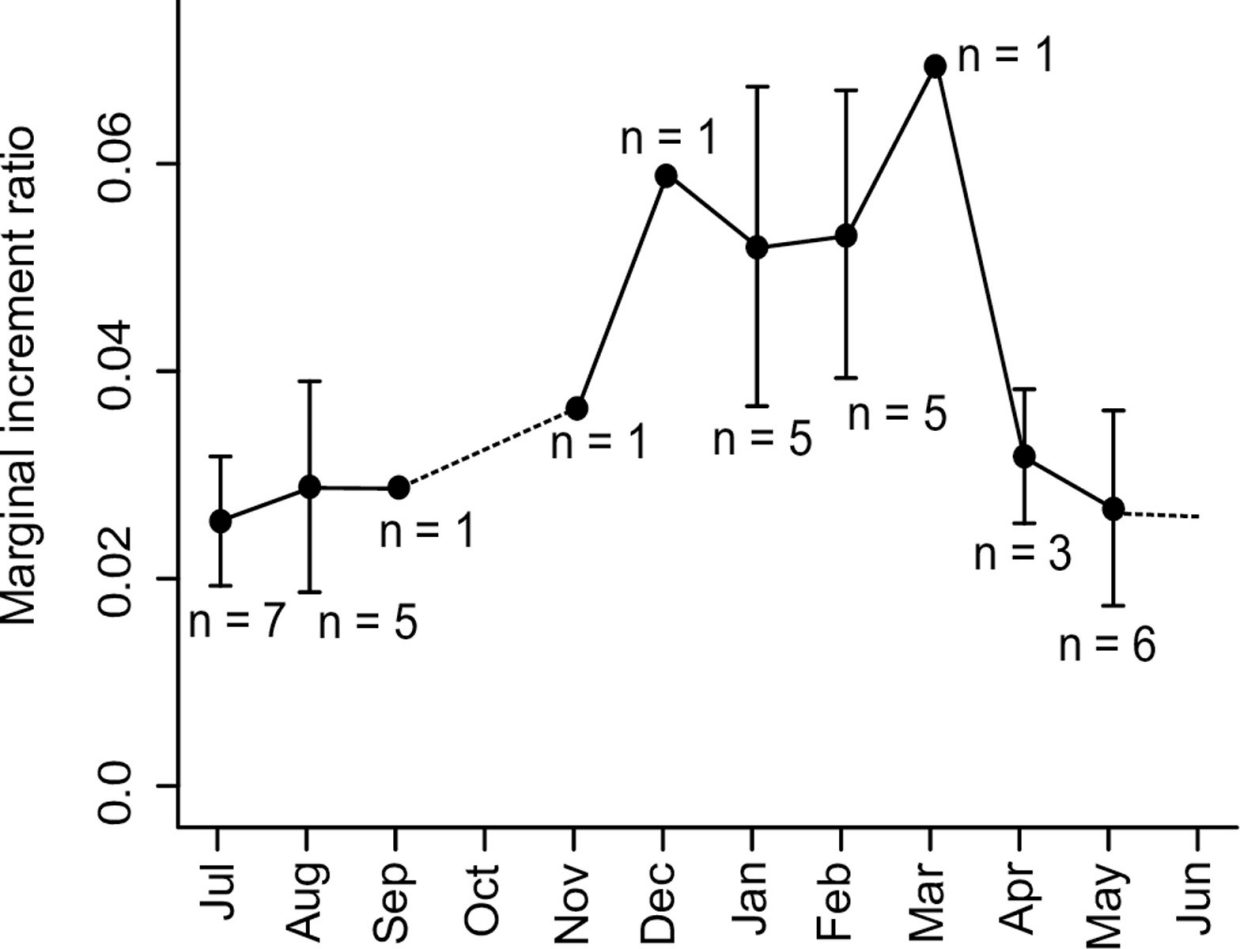
Monthly variation in the mean marginal increment ratio for *Brachyhypopomus bennetti* (n = 35). Here, marginal increment ratios were calculated by (R_C_-R_L_)·(R_L_)^-1^ where R_C_ = the radius of the otolith and R_L_ = the distance from the center of the otolith core to the center of a single dark annulus. Black dots and error bars represent the monthly mean +/- 1 standard deviation. Dotted lines connect months with missing intervening monthly means (See S5 Appendix for supporting data).

Marginal increment analysis of the floodplain species *B*. *bennetti* (Fig 7, S5 Appendix) revealed that the highest marginal increment ratios occur in December-March (during the peak of the breeding season) and revealed a marked reduction in marginal increment ratios between April and September (during a low-water breeding hiatus, see ‘Seasonal variation in reproductive status’, below). This pattern verifies that opaque band formation occurs from the middle to the end of the breeding period for floodplain *Brachyhypopomus*. Since there is a sinusoidal pattern of marginal increment ratio during the year, we concluded that opaque bands of *B*. *bennetti* are true annuli sensu Campana [58]. For all species we interpret the wide opaque bands to represent a period of reduced growth during the breeding period, which we presume arises from a diversion of somatic resources to gonadal development and other reproductive functions [59–61].

Based on the results of our size-frequency and otolith morphology analyses, we conclude that *B*. *bennetti*, *B*. *sullivani*, *B*. *verdii*, and *B*. *walteri* have a one-year lifespan, with reproduction during a single protracted breeding period followed by death. In contrast we concluded that *B*. *beebei* has a two-year lifespan with a single protracted annual breeding period in both the first (0+) year-group and a second (1+) year group; we expand this argument in the Discussion.

We also compiled evidence to suggest that three of the rarer species, *B*. *benjamini*, *B*. *flavipomus*, and *B*. *regani* also have one-year life histories. From samples taken during the breeding period only (here data for the floodplain species was collected during a later expedition to the study area), FMMs recovered single size-groups of adults (S6 and S7 Appendices). Also, otolith preparations of late-breeding season Stage 4 individuals in these three species revealed the occurrence of only one dark annulus (n = 2 of 2 preparations for *B*. *benjamini*: n = 2 of 2 preparations for *B*. *flavipomus*; n = 1 of 1 preparation for *B*. *regani*) (S4 and S8 Appendices).

Likewise, we propose that *B*. *brevirostris*, which is rare in the study area (but a common eurytopic species elsewhere [9]), also has a multi-year iteroparous life history. From samples taken during the breeding period in a later expedition to the study area, FMMs recovered two size-groups of adult specimens (corresponding to individuals in the > 0 ≤ 150 mm LEA, and > 150 mm ≤ 208 mm LEA size-groups, respectively) (S6 and S7 Appendices). Also, we reported two distinct dark annuli in otolith preparations of a single late-breeding season Stage 4 female belonging to the large size group but a single annulus in a Stage 4 female belonging to the smaller size group (S4 and S8 Appendices).

### Seasonal changes in habitat structure and species distributions

#### Floodplain lakes

During the high-water period, floodplain species of *Brachyhypopomus* formed a common component of the fish fauna in floating meadow stands, mirroring observations for other Amazonian floodplains [57, 75]. We concluded that reproduction occurs within the root mat because during the breeding season, our fish finder often detected male-female dyads exhibiting intense synchronized ‘chirp’ modulations of their EODs, which have been well-described in the genus [19, 76, 77]. We never observed courtship behavior outside the floating meadows. Some *Brachyhypopomus* and other gymnotiforms moved into flooded forest to feed in submerged leaf litter along the land-water interface during the early rising-water period (we did not include these habitats in our quantitative CPUE survey), but at high water we found very few gymnotiforms in the flooded forest in comparison to floating meadows in the lakes. During the high-water period the bottom layers of the floodplain water column were completely anoxic (see similar observations in Henderson et al. [27] and Crampton et al. [78]), and our fish finders detected no electrical activity at all at depths exceeding 1 m below the surface. During the low-water period we observed a marked contraction of the area of macrophyte stands in floodplain lakes, which mostly became stranded on beaches or vegetation around the lakes (Fig 1C). At this time of the year *Brachyhypopomus* were confined to remnant patches of floating macrophytes in deep portions (2–3 m) of the lakes, or to shallow vegetation-choked pools in low-lying terrain. During the low-water period, we observed a substantial increase in the density of piscivorous predators that consume gymnotiforms (based on stomach content analyses)–notably *Pygocentrus nattereri*, *Hoplias malabaricus*, *Hydrolycus* spp., and *Rhaphiodon vulpinus* (unpublished results based on non-quantitative sampling in floodplain lakes and channels of the nearby Pacaya Samiria Reserve). At low water there is also a substantial increase in the density of piscivorous birds (primarily herons), aquatic snakes, and caiman in whitewater floodplain systems.

#### Terra firme systems

Although not subject to the influence of river-floodplain flooding, terra firme streams flood adjacent depressions and the lower reaches of feeder streams in response to local rainfall, forming extensive temporary shallow swamp systems [79]. Our fish-finder surveys indicated that some individuals of all species entered these shallow swamp systems during their period of nocturnal activity. The swamps reached their greatest extent during the annual rainy season. During the dry season they periodically contracted into small pools but were rapidly recharged by rainstorms, which caused the streams to exhibit a brief (< 24 hrs.) increase in level by up to ca. 0.4 m. Three species, *B*. *beebei*, *B*. *verdii*, *and B*. *walteri* were commonly encountered in these swamps. However, all individuals returned to the mains stream courses when the swamps dried out. We noted that these three species both fed (based on observations of stomach contents) and reproduced (based on the detection of courting dyads, and the presence of post-larval and juvenile fish) in the swamps. Two species, *B*. *benjamini* and *B*. *sullivani* were also occasionally found in the terra firme swamps (usually in the lower courses of feeder streams) but closer to the main stream channels (typically < 5 m). During the low-water period, as seasonal swamps contracted, we observed an increase in the density of piscivorous predators that consume gymnotiforms. These included *Hoplias malabaricus*, *Hoploerythrinus unitaeniatus*, *Acestrorhynchus* spp., *Rhamdia* sp., and *Electrophorus varii* (unpublished observations from general ichthyological sampling of streams in the study area). Other likely predators of gymnotiforms in terra firme streams of the upper Amazon include herons, water snakes, dwarf caiman (*Paleosuchus* spp.) and the Neotropical otter *Lontra longicaudis*.

### Seasonal variation in reproductive status

**Fig 8:**
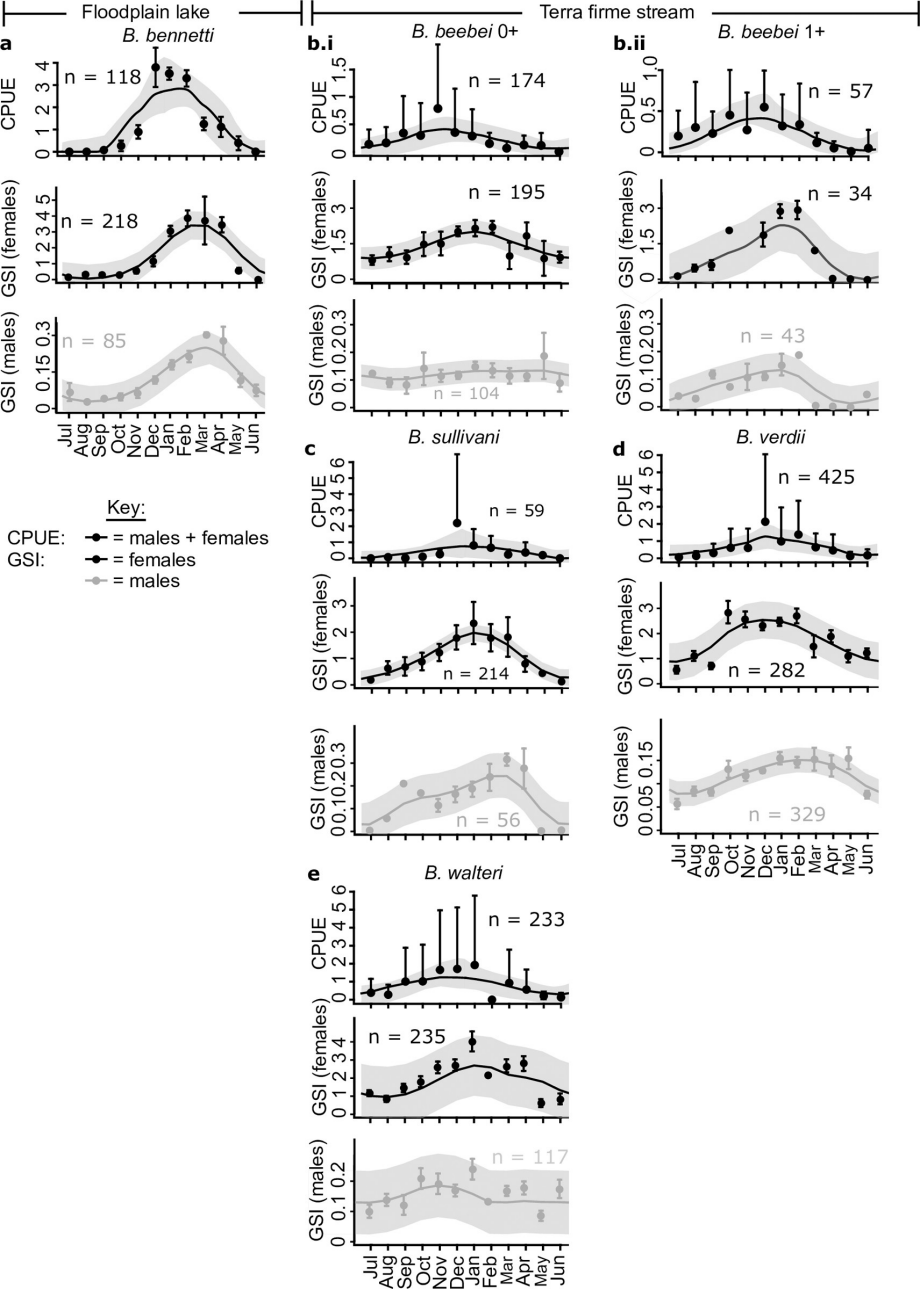
Circannual variation in capture per unit effort abundance of spawning capable adults (gonad Stages 3+4) and gonadosomatic index for all sexed individuals (females and males at gonad Stages 2–5) of five species of *Brachyhypopomus*. Dots and error bars represent monthly means with +/- 1 standard deviation (only one error bar is provided where plot is congested). Black line and grey shaded area represent spline fit with upper and lower 95% spline confidence intervals.

**Fig 9:**
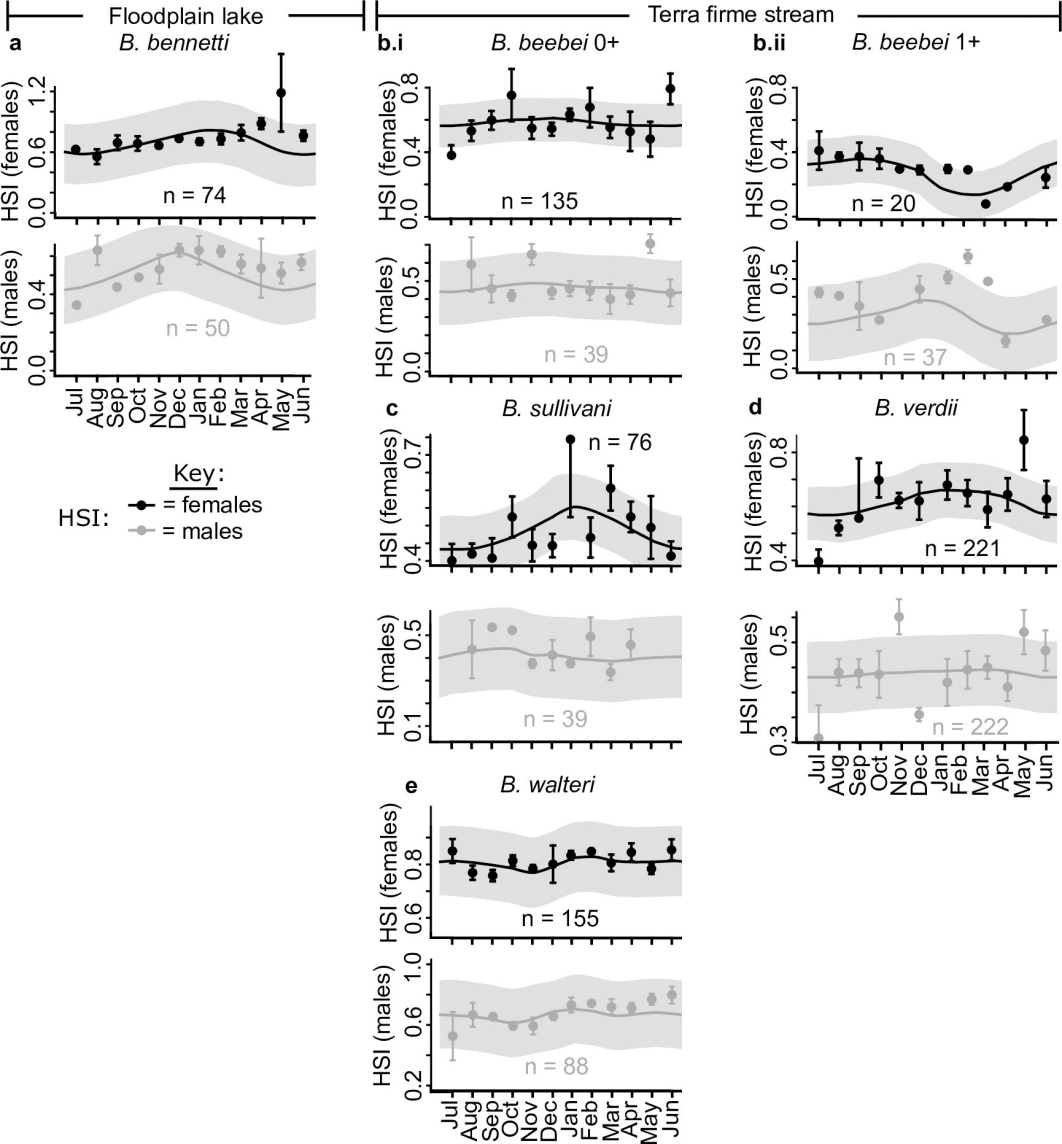
Circannual variation in hepatosomatic index for all sexed individuals (gonad Stages 2–5) of five species of *Brachyhypopomus*. See Fig 8 caption for further details.

All species exhibited a single prolonged annual breeding season, the peak of which coincides with the period of rising-water and high-water in the floodplain, and the late dry-season and early rainy season in the terra firme streams. This pattern was evident from circannual variation in the CPUE of Stage 3 and 4 individuals (Fig 8), female and male GSI (Fig 8), and female and male HSI (Fig 9), all of which exhibited a sinusoidal oscillation with an annual period (see spline fit and 95% spline confidence intervals). In most cases the maxima of the spline fit to the CPUE of Stage 3 and 4 individuals preceded the maxima of the GSIs and HSIs. The minima of the spline fits to the CPUE of Stage 3 and 4 individuals corresponded in all species to a point between the second half of May and first half of June, indicating a common habitat-wide end to breeding in both floodplain and terra firme habitats (to represent events preceding this point we used a July-June abscissa sequence in Figs 8 and 9). However, the shape of the spline-fit to the CPUE of Stage 3 and 4 individuals indicates that the floodplain species *B*. *bennetti* has a shorter and more demarcated breeding season than the terra firme species, some of which (e.g. *B*. *beebei* in both year-groups) breed almost year-round with only a brief hiatus.

In the iteroparous species *B*. *beebei* we noted a larger circannual variation in mean GSI for the 1+ year group (Fig 8B.ii) than in the 0+ year group (Fig 8B.i), especially for females (see amplitude of spline-fit periodicity). This pattern in part reflects lower GSI values for the final four months of the breeding season in the 1+ year group than in the 0+ year group.

### Seasonal variation in hepatosomatic index

The females of most species in the study area exhibited a sinusoidal annual oscillation in HSI, approximately in tandem with GSI, indicative of a relative enlargement of the liver during the breeding period relative to the non-breeding period (Fig 9). For teleost fishes in general, the liver performs multiple functions, including the production of enzymes for digestion, nitrogen catabolism, and detoxification, as well as temporary lipid storage [80, 81]. In female teleosts, the liver additionally serves as the primary site of synthesis for the egg yolk protein precursor vitellogenin [81]. Because of the key role of the female fish liver in oogenesis, we were therefore unsurprised to observe increases in female HSI in approximate tandem with increases in GSI across all species. However, we also noted an elevation of HSI during the early parts of the breeding season in the *males* of *B*. *bennetti*, and 1+ group *B*. *beebei*, suggesting that the liver may play a role in supporting male reproductive functions in these species via the depletion of lipid reserves.

In both sexes of the iteroparous species *B*. *beebei*, we reported a more extreme amplitude of HSI oscillation for the 1+ group than the 0+ year group (Fig 9B), mirroring the observation for GSI (Fig 8B). We also observed substantially higher HSI values in both sexes of the *B*. *beebei* 0+ year group than the 1+ year group (2-tailed t-test for females, t = 4.3, *p* = 2E-5; for males, t = 3.7, *p* = 3E-3). This disparity was especially pronounced towards the end of the breeding season, following a decline in the HSI of the 1+ group, but not the 0+ group (compare splines in Fig 9Bi to Fig 9Bii). We return to the implications of these observations in the Discussion.

### Oocyte size distributions and counts

**Fig 10:**
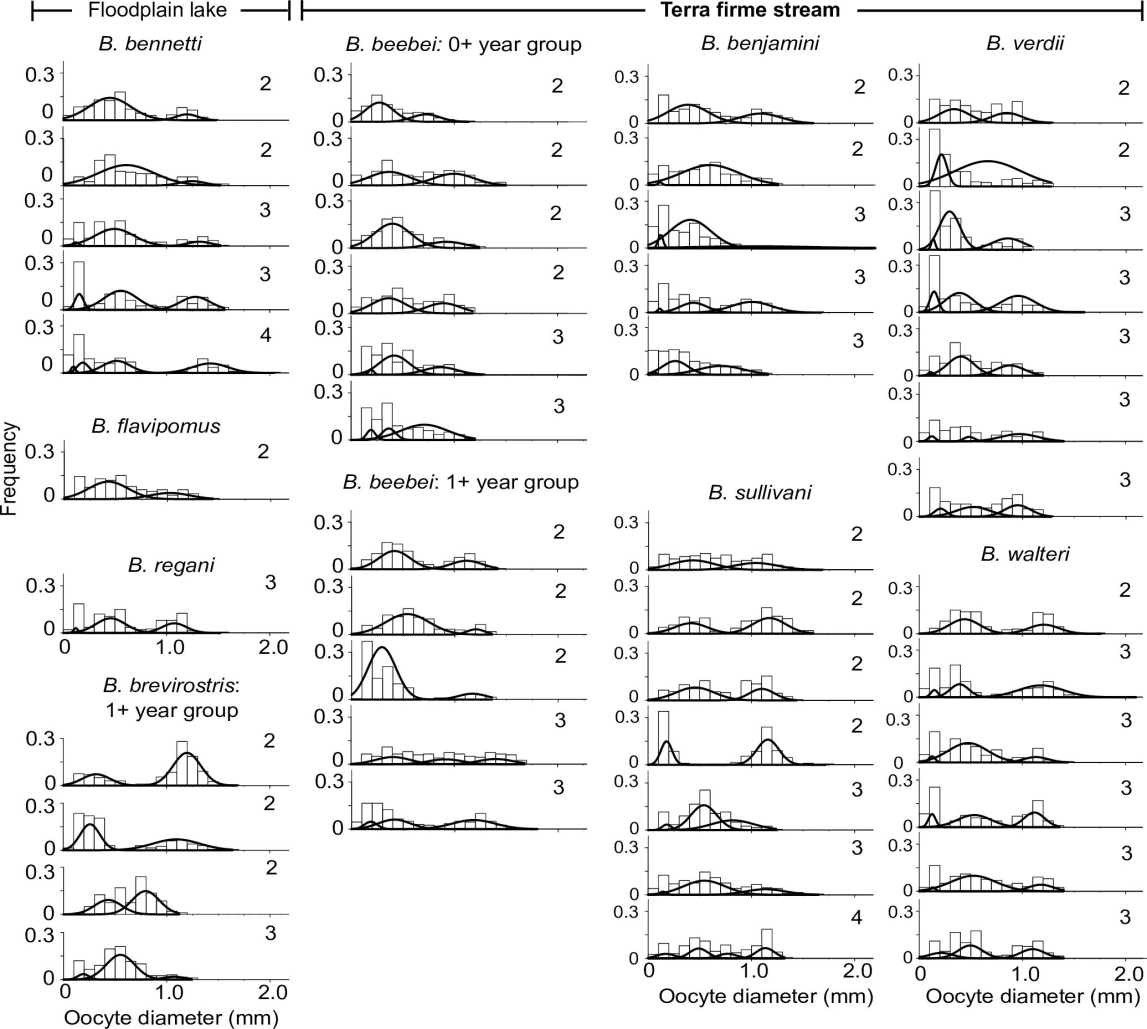
Histograms of oocyte diameters in spawning capable females of nine species of *Brachyhypopomus* in the active spawning subphase (Stage 4). Dashed curves represent Gaussian distributions fitted by Finite Mixture Models to show modal size groups. The number of size modes is reported to the right of each histogram.

Analyses of oocyte size-frequency distributions (Fig 10) reported 2–3 (and rarely 4) modal oocyte size groups per individual in most individuals of all species. These patterns are consistent with a fractional spawning strategy in which oocytes mature continuously and are released in successive batches separated by recovery periods [48, 68, 69]. Bakos [82] reports the oocytes of freshwater fishes to swell 1.6–1.7 times in diameter immediately after spawning. When adjusted for such post-spawning swelling, our measure of the mean oocyte size in the largest oocyte category for *B*. *brevirostris* (1.1 mm in the ovary, corrected to ca. 1.8 mm) corresponds precisely to the 1.8 mm diameter reported for the same species by Kirschbaum & Schugardt [83]. This confirms that the largest oocyte class in our size-frequency distributions is the one released in spawning events.

For the ovaries reported in in Fig 10, the estimated number of oocytes in the largest oocyte group varied as follows: In floodplains–*B*. *bennetti* (98–300, mean = 199, n = 5); *B*. *flavipomus* (156, n = 1); *B*. *regani* (215, n = 1); *B*. *brevirostris* 1+ year group (94–195, mean = 144, n = 4); in terra firme streams–*B*. *beebei* 0+ group (38–457, mean = 187, n = 6); *B*. *beebei* 1+ year group (45–356, mean = 229, n = 5); *B*. *benjamini* (84–243, mean = 172, n = 5); *B*. *sullivani* (122–195, mean = 156, n = 7); *B*. *verdii* (34–147, mean = 82, n = 7); *B*. *walteri* (127–208, mean = 157, n = 6).

### Parental care

We located three small aggregations of juvenile *Brachyhypopomus beebei* (10–20 individuals in the 20–40 mm LEA range within a ca. 40 x 40 cm area). These were hidden among submerged leaf litter in shallow terra firme swamps. We also found two similar aggregations of *B*. *bennetti*, of similar size, in root clumps of *Eichhornia crassipes* in floating macrophyte stands (here we include ca. 75 hrs. of additional fish finder surveys by WGRC in floodplains of the nearby Pacaya Samiria Reserve). However, we did not find adult conspecifics in the immediate vicinity of these aggregations. Our observations resemble reports of small “crèches” of *B*. *beebei* in rainforest streams of French Guiana by Westby [84]. Because these were the only observations of larval aggregations of *Brachyhypopomus* in over 1000 hrs. of fish-finder surveys, we conclude that parental care is absent in *Brachyhypopomus* from our study area.

## Discussion

### Breeding periods

Our observations of circannual variation in the CPUE of fully reproductive fish, and matched circannual variation in GSI and HSI, are consistent with a single protracted annual breeding season in all species of *Brachyhypopomus*. This breeding season is approximately synchronized to the rising and high-water period in the floodplain systems, and to the late dry season and rainy season in the terra firme systems. These patterns are similar to those observed in several other studies of floodplain species, e.g. [85–88], and rainforest stream species, e.g. [89–92], and are consistent with selection to synchronize reproduction to the period of the year most favorable to offspring growth [93].

In large Neotropical floodplain systems, small fishes are known to experience a major reduction in habitat availability during the low-water period [24, 94], and are subject to much higher levels of predation [25, 95] than during the high-water period. Although terra firme systems exhibit a small magnitude of flood amplitude (< 0.5 m) in comparison to river-floodplain systems (up to 16 m) [27], Junk [96] estimates that the intermittent and seasonal flooding of low-order terra firme streams results in the shallow inundation of a remarkable one million km^2^ of the Amazon forest (some 18% of the entire area of the basin). These shallow swamps likely play an important role in the dynamics of terra firme stream fish communities by functioning as seasonal feeding grounds and refuges from hypoxia intolerant predators [2, 29, 79, 97]. Due to the decomposition of submerged forest leaf litter, terra firme swamps are hypoxic and therefore populated primarily with species that breathe air or have other means of tolerating low levels of dissolved oxygen [79, 98]. In summary, our study suggests that the timing of reproduction in terra firme species of *Brachyhypopomus*, as well as in floodplain species, is closely tied to a seasonal expansion and contraction of habitat, and probably also to corresponding variation in predator densities.

### Iteroparous versus semelparous life histories

Based on our observations of size-frequency distributions, and otolith morphology, we divided *Brachyhypopomus* species into two life-history categories, with no intermediates.

1. *Semelparous species with an approximately one-year (annual) lifespan followed by death*. This category includes the floodplain lake species *B*. *bennetti*, *B*. *flavipomus*, and *B*. *regani*, and the terra firme stream species *B*. *benjamini*, *B*. *sullivani*, *B*. *verdii*, and *B*. *walteri*. These species appear to breed repeatedly (via fractional spawning events in females) during a single protracted breeding period, which is followed soon after by death. Kirkendall & Stenseth [32] refer to this type of life history as ‘uniseasonal iteroparity’. As described in the Introduction, we follow others in classifying the uniseasonal-iteroparous strategy of annual *Brachyhypopomus* species as a form of semelparous life history (i.e. with a single reproductive period preceding death), e.g. Gavassa & Stoddard [34], Gavassa et al. [21], and Sinnett & Markham [35]. Kirkendall & Stenseth [32] and Roff [33] likewise concluded that animals with uniseasonal iteroparity belong near the semelparous end of a theoretical continuum between semelparity and iteroparity.
  Previous studies of two congeners: *B*. *gauderio* by Silva et al. [16] (see Fig 5 therein) and *B*. *occidentalis* by Hagedorn [19] appear to report a similar (uniseasonal iteroparous) form of short-lived semelparous life history, based on the disappearance of adult individuals following an annual breeding season; Gavassa and Stoddard [34] and Kirschbaum and Schugardt [74] reached similar conclusions regarding the findings of Silva et al. [16] and Hagedorn [19]. Both Silva et al. [16] and Hagedorn [19] describe post-breeding mortality as one of several possible explanations for the disappearance of adults following the breeding season–including dispersal (by migration or wash-out), and differential predation. However, these alternative explanations were mostly ruled out in Hagedorn’s study [19], which also cited Heiligenberg (pers. comm.) for direct observations of post-spawning mortality in a congener. In our study, because we sampled all available *Brachyhypopomus* habitats, we can categorically rule out dispersal as an explanation for the disappearance of adults following the annual breeding season. Higher predation on post-reproductive adults is possible but would in any case be expected in individuals exhausted by breeding and therefore constitutes part of a phenomenon of terminal post-reproductive mortality. Silva et al. [16] noted that captive *B*. *gauderio* can live for more than one year and breed in two successive austral summers. Nonetheless, although the observation raises the obvious conclusion that life history may be phenotypically plastic in this species, the tendency for aquarium-kept fish to live and continue breeding for considerably longer than wild conspecifics has been noted in multiple freshwater fish species (see review by Beverton & Holt [99]). Therefore, evidence for multi-year lifespans from captive fish should not be used to conclude similar lifespans in the wild.
2. *Iteroparous species with a ca*. *two-year lifespan*. This category comprises two species from our study area: *B*. *beebei* and *B*. *brevirostris*. The former (for which obtained a much larger dataset) exhibits a single annual breeding season in both a 0+ and a 1+ year group (corresponding to individuals in the > 0 ≤ 115 mm LEA, and > 115 mm ≤ 210 mm LEA size-groups, respectively), and exhibits complete post-reproductive mortality in the 1+ group. Kirkendall and Stenseth [32] refer to this type of iteroparous life history as ‘multiseasonal iteroparity’. We found no evidence for a third size-group in this species indicative of survival to a third year. Averaged over all sample sites, we captured 423 breeding and non-breeding *B*. *beebei* (excluding specimens < 30 mm TL) in the 0+ group but only 93 in its 1+ group, suggesting that approximately only one in four individuals (> 30 mm TL) survive from the 0+ year group to the 1+ year group in this species.

Multi-year life histories have previously been reported for several gymnotiform fishes, including *Eigenmannia sp*. [100], *Apteronotus leptorhynchus* [101], and *Gymnotus* sp. and *Sternopygus* sp. [74]. Outside the gymnotiforms, the life history strategy most commonly reported in Neotropical fishes is iteroparity. Examples of species which live for multiple years and exhibit multiple breeding seasons include *Arapaima gigas* [102, 103], *Colossoma macropomum* [104], some migratory pimelodid catfishes [67, 105], *Pygocentrus nattereri* [62], and *Calophysus macropterus* [106]. Predominantly two-year lifespans have been reported in *Poecilia reticulata* [107] and *Symphysodon haraldi* [88] (although both species live and breed for longer in aquaria), but we are unaware of other Neotropical species reported to have two-year life histories. One-year lifespans are well-known in some cyprinodontiforms taxa [108] but have yet to be described from other orders of Neotropical fishes, including among the many groups of small or miniaturized fishes sensu Weitzman and Vari [109], where the phenomenon is most likely to have evolved.

We were unable to formulate plausible alternative explanations for our conclusion that some *Brachyhypopomus* species have (semelparous) annual lifespans with a single reproductive period followed by post-breeding mortality, while others have (iteroparous) two-year lifespans with post-breeding mortality deferred to the end of the second breeding season. For instance, it is unlikely that interspecific or intraspecific variation in growth rates could account for the simultaneous disparities in size-frequency distributions and otolith growth annuli in our study system. Our size-frequency distributions and otolith growth-annulus assessments (including marginal increment analyses) are entirely congruent in inferring maturation within the first year for all species in our study area, and in substantiating a second year-group only for *B*. *beebei* and *B*. *brevirostris*. Maturation within the first year has been previously demonstrated in *B*. *gauderio*, which is known to reach maturity within 4–5 months of hatching [74], despite considerable variation among individuals in growth rates [110] (Fig 4, therein). Further, both annual semelparous and multi-year iteroparous life histories occurred in oligotrophic terra firme streams as well as in nutrient-rich whitewater lakes (where growth rates could be elevated due to higher ecosystem productivity). Therefore, variation in growth rate related to nutrient availability cannot explain our observations of life history variation in *Brachyhypopomus*.

### Spawning strategies

All species of *Brachyhypopomus* in our upper Amazon study system exhibit fractional spawning. We assume that this is the ubiquitous condition in the genus because it has also been inferred from analyses of oocyte size distributions in several other congeners, including *B*. *bombilla* [14], *B*. *draco* [13], *B*. *gauderio* [15, 17], and *B*. *occidentalis* [19], or from direct observations of spawning in captive fish–including in *B*. *pinnicaudatus* and *B*. *brevirostris* [74], and *B*. *gauderio* [111]. Fractional spawning in fishes is typically viewed as a bet hedging strategy to spread out offspring mortality associated with recruitment time across multiple spawning events, and to reduce the impact of entirely failed spawning events on maternal fitness [40, 68]. The phenomenon is widespread in Neotropical fishes, including in other gymnotiforms, e.g. *Gymnotus* [112] and *Eigenmannia* [113], as well as in many cichlids, small-bodied characiforms, and siluriforms [9]. In contrast, total spawning in Neotropical fishes is mostly restricted to migratory riverine characiforms and large siluriforms [9].

Although we reported 2–3 groups of oocytes in the ovaries of most *Brachyhypopomus*, we are unsure of how many and how often these eggs are released. Kirschbaum and Schugardt report spawning on successive nights in *B*. *pinnicaudatus* and *B*. *brevirostris*, with approximately 70 and 50 eggs per spawning. *Brachyhypopomus gauderio* spawn every 3–8 days when releasing a ripe batch of eggs [73]. Given these behavioral observations and given variation in the total number oocytes in the largest oocyte group (see Results: Oocyte distributions and counts), we estimate that the females of most *Brachyhypopomus* species release 50+ eggs on multiple nights during the release of a ripe batch of oocytes, followed by a period of unknown duration before the next batch matures.

We are also unsure of how many batches of oocytes are produced during the breeding season of *Brachyhypopomus*, primarily because our study is uninformative of whether *Brachyhypopomus* have indeterminate spawning, sensu Murua [114], whereby new vitellogenic oocytes are constantly recruited in the ovary during the breeding period (in these cases the number of batches of eggs in an ovary may not reflect the total number of batches that will be produced and potentially laid during the breeding season). The alternative, determinate spawning, in which a female produces a finite supply of oocytes, may be less likely in *Brachyhypopomus* because this strategy is relatively uncommon in fractional spawning tropical species [48, 115].

### Parental care and brood hiding

We concluded that parental care is absent in the *Brachyhypopomus* species of our study system. The phenomenon has not previously been reported in the genus, except by Giora [15], who reported the presence of adult males with aggregations of larval *B*. *gauderio*. However, aquarium studies of the same species report no parental care after a lengthy spawning process during which females select oviposition sites for individual eggs in disparate sites; the eggs are fertilized by an accompanying male but abandoned thereafter [73]. Similar ‘brood hiding’ behavior sensu Balon [116], without parental care, has been documented in *B*. *brevirostris* and *B*. *pinnicaudatus* [74]. Parental care is, however, known from several other gymnotiforms, including in *Gymnotus carapo* [72], which exhibits mouth brooding of newly hatched larvae [74], *G*. *mamiraua* [72], *Electrophorus ‘electricus’* (probably *E*. *varii*), the males of which form bubble nests [117], *Sternopygus macrurus*, in which males mouth brood eggs [74], and *Eigenmannia trilineata* [118].

### Life history and habitat

We hypothesized (see Introduction) that specialization to floodplain systems and terra firme systems should predict semelparous and iteroparous life histories, respectively. This hypothesis is based on three premises: (1) Williams [36] hypothesized that greater mean adult mortality, relative to offspring mortality (which is usually controlled by predation, disease, and food availability) is expected to favor shorter lives with fewer reproductive events–i.e. strategies near the semelparous end of the semelparity-iteroparity continuum. Support for this hypothesis has come from studies of the effects of predation on guppies [119] and sticklebacks [120]. Here we assume that fishes in floodplain systems exhibit higher levels of predation-induced mortality (at least during the dry-season) than in terra firme systems, largely due to the greater seasonal variation in water volume in floodplains [23, 25]. (2) Elevated variance of offspring survival rate associated with high season-to-season environmental stochasticity in unstable environments is also expected to favor shorter lifespans and higher intrinsic rates of increase [1]. By most criteria, floodplain systems exhibit more environmental stochasticity than terra firme stream systems. For example, they are subject to more extreme circannual variation in flood amplitude, substrate availability, and predator densities than terra firme systems [24, 27, 28]. Also, shallow floodplain lakes dry out entirely in years with below average water level [121]–resulting in stochastic population bottlenecks for small fish species [1, 122]. (3) Studies have demonstrated that high ecosystem productivity is associated with shorter lifespans and fewer reproductive events (i.e. strategies approaching semelparity), while low ecosystem productivity can favor transitions to longer life spans with more reproductive events (i.e. strategies approaching iteroparity) [37, 120, 123]. Neotropical floodplains (especially nutrient-rich whitewater systems) typically have higher autochthonous and allochthonous productivity than small oligotrophic forest stream systems [2, 124] and should therefore favor semelparous strategies.

Nonetheless, the ecological distributions of semelparity and iteroparity in our study system provided no obvious support for this hypothesis. Seven species are semelparous, of which three are specialized to floodplains (*B*. *bennetti*, *B*. *flavipomus*, and *B*. *regani*), and four are specialized to terra firme streams (*B*. *benjamini*, *B*. *sullivani*, *B*. *verdii*, and *B*. *walteri*). Two species, *B*. *beebei* and *B*. *brevirostris* are iteroparous, both of which are eurytopic (occupy both floodplains and terra firme streams), although in the study area *B*. *beebei* was more common in streams and *B*. *brevirostris* in floodplains (elsewhere in the Amazon *B*. *beebei* and *B*. *brevirostris* commonly occur in both floodplain and terra firme systems).

### Life history and age-regulated terminal investment

The distribution of semelparity and iteroparity across *Brachyhypopomus* in our study system does not appear to have been strongly influenced by selective forces related to habitat occupancy. Instead, we argue that semelparity and iteroparity represent alternative evolutionary stable strategies (ESSs) for allocating resources to reproduction versus to somatic growth and maintenance at different life stages. Annual semelparous species effectively exhibit a go-for-broke strategy, in which resources are channeled from growth and maintenance to current reproduction in their single, final breeding season, ensuring death by reproductive exhaustion as a byproduct [21, 40]. In contrast, iteroparous species with a two-year lifespan trade-off survival and reproduction across two breeding seasons. In these species we hypothesized an age-specific pattern of terminal investment sensu Williams [43] in which resources are diverted from the soma to reproduction as residual reproductive value declines to zero in the terminal 1+ group, but a moderation of reproduction during the 0+ group in favor of growth and somatic maintenance–thereby increasing prospects for a second reproductive season [43, 125].

We made two sets of observations consistent with the predicted pattern of reproductive restraint in the 0+ group of *B*. *beebei* relative to the 1+ group. First, we documented that the HSI of male and female *B*. *beebei* is substantially higher in the 0+ group than the 1+ group and declines more rapidly and to lower levels during the breeding season of the 1+ group than of the 0+ group–suggesting that proportional liver size is conserved in the first year of life but depleted to sustain reproduction in the terminal 1+ breeding season. These observation provide strong support for our prediction (see Introduction) that species with a two-year life history might switch from an income breeding strategy sensu Jönsson [45] in the 0+ group to an unsustainable capital breeding strategy in the 1+ year group [45, 126].

**Fig 11:**
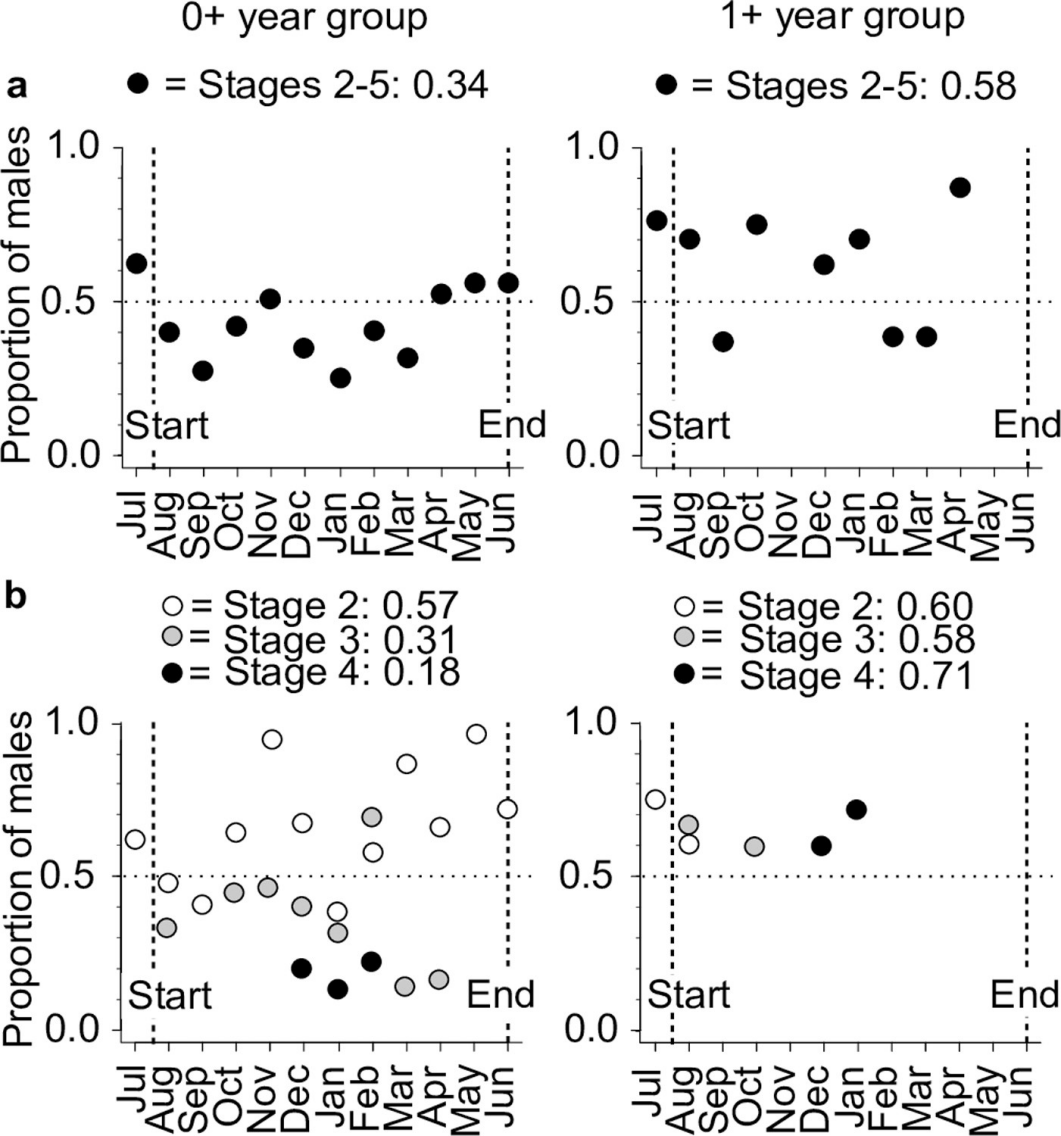
Variation in sex ratio for the 0+ and 1+ year groups of *Brachyhypopomus beebei*. a. Sex ratio for all sexed individuals (gonad Stages 2–5). b. Sex ratio of individuals at successive gonad stages (2 through 4). For a. and b. results for a given month are provided only where n individuals per category ≥10. Ratios reported at the top of each plot refer to all 12 months of the study combined.

The second observation consistent with a pattern of relative reproductive restraint in the 0+ group is that the ratio of male to female *B*. *beebei* changes substantially between the 0+ and 1+ year groups (see Fig 11). For all adults that we were able to assign to sex (i.e. gonad Stages 2–5), we observed a shift from a female-biased sex ratio in the 0+ year group (n males/n females = 0.34) to a slightly male-biased sex ratio in the 1+ year group (n males/n females = 0.58) (Fig 11A). Moreover, in the 0+ year group, the female bias increased as a function of increasing maturation–with individuals at Stage 2 exhibiting a slight male bias, but individuals at Stages 3 and 4, respectively, exhibiting an increasing female bias. In contrast, in the 1+ year group, the male bias was common to all stages of maturity (Fig 11B). We postulate that these changes in sex-ratio arise because 0+ males exhibit a form of reproductive restraint in which many individuals arrest or delay gonadal maturation to avoid (or restrict) breeding in their first year and instead concentrate on feeding and somatic growth. This strategy would increase the chance of males surviving to their second breeding season as well as increase their body size in the second season. In fishes, body size is typically positively correlated to fecundity and mating success [127–129], and in *Brachyhypopomus*, male reproductive success is known to be elevated in individuals with large body size (and high EOD amplitude), including in mate choice [130] and male-male dominance conflicts [131, 132]. Moreover, even a small body size difference between rival males may predict a greater chance of reproductive success, because EOD peak-to-peak EOD amplitude increases as an exponential rather than linear function of total body length [133]. Therefore, selection for larger body size in the 1+ group via a moderation of reproductive effort int the 0+ group is expected to be especially strong in males.

## Conclusions

**Fig 12:**
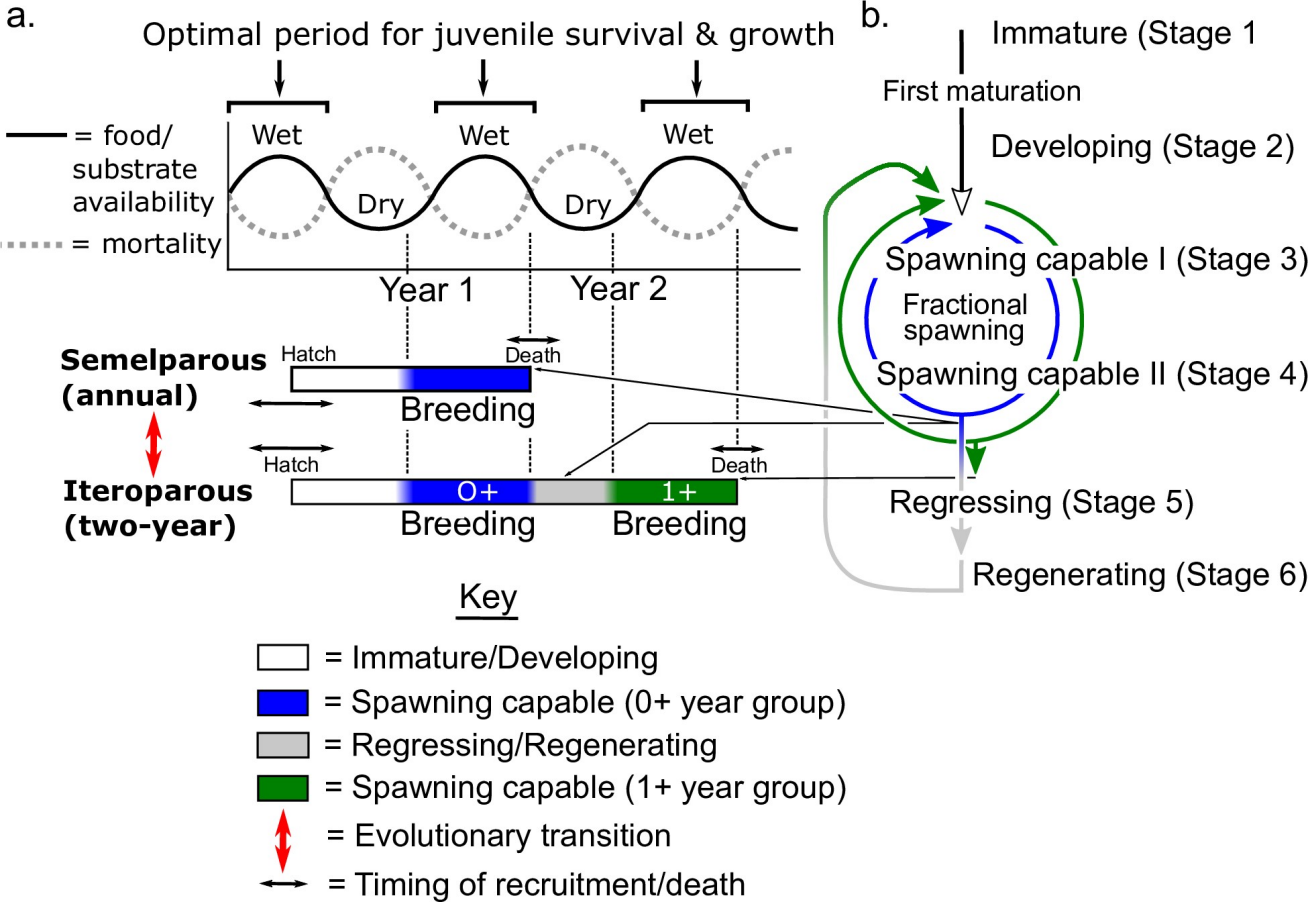
Summary conceptual models of semelparous (annual) and iteroparous (two-year) life histories as alternative evolutionarily stable strategies in seasonally variable Amazonian habitats. a. Seasonal variation in predator-induced mortality and food/substrate availability is expected to drive an adaptive pattern of reproduction during a single prolonged annual breeding season period, corresponding to the wet season (= rainy season/high water) period of optimal survival and juvenile growth. During the breeding period, females reproduce in multiple fractional spawning events. Species with iteroparous two-year lifespans add an additional year to their lifespan and delay post-reproductive mortality from the end of the 0+ year group to the end of a second, 1+ year group. Evolutionary transitions between semelparity and iteroparity do not appear to be correlated to habitat (floodplain versus terra firme streams) in upper Amazon *Brachyhypopomus*. The iteroparous species *B*. *beebei* exhibits a pattern of reproductive restraint in the 0+ year group relative to the 1+ year group consistent with terminal reproductive investment. Reproductive restraint in the 0+ group may increase the probability of survival through the dry-season period of elevated mortality (individuals that survive to the 1+ year group are expected to benefit from increased fecundity and mating success than 0+ individuals). Note: life spans of both semelparous and iteroparous species likely exhibit variation related to the time of recruitment and death (see horizontal double arrow lines). b. Conceptual model of reproductive maturational stages, adapted from Brown-Peterson et al. [48], showing cyclical progression of maturational stages for a 0+ year group (of annual semelparous and two-year iteroparous species) in blue, and a 1+ year group (for two-year iteroparous species only) in green. Spawning capable (Stage 3 and 4) females cycle back and forth (see circular arrows) between Stage 3 (subphase I–not actively spawning) and Stage 4 (subphase II–actively spawning) as successive batches of oocytes are released in fractional spawning events, each of which lasts several nights.

The monographic multi-species approach we adopt in this study provides the first comparative examination of life history variation within a monophyletic group of closely related Neotropical fishes. Some of the patterns we reveal here are common to other Neotropical fishes–including synchronization of a single protracted annual breeding event to an optimal period for offspring growth, and fractional spawning. Our application of CPUE-based size-frequency analyses (based on large sample sizes of individuals captured through an entire year) combined with otolith growth-increment analyses allowed us to assign all species in our study area to either an annual semelparous life history, or to a two-year iteroparous life history. We found no correlation between habitat occupancy and the distribution of semelparity and iteroparity in *Brachyhypopomus*, despite predictions from life history theory. Instead we conjecture that semelparous and iteroparous life histories represent alternative ESSs, with similar success in a variety of seasonal Neotropical aquatic habitats; see conceptual models in Fig 12. We also noted that several aspects of the reproductive biology of the single common iteroparous species *B*. *beebei* are consistent with theoretical expectations for age-regulated terminal reproductive investment.

Having established a baseline understanding of the reproductive life history strategies of *Brachyhypopomus*, we hope that our upper Amazon study system will serve as a new and productive model system for exploring life-history trade-offs in energy allocation. Unlike in most animal communication systems, energy investment in the electric signals of gymnotiforms can be quantified by readily measurable aspects of the EOD, such as amplitude and repetition rate [22, 134]. Our system is therefore uniquely amenable to further investigations of how energy is allocated to growth and somatic maintenance versus to reproductive communication signaling through an animal’s life, and as a function of residual reproductive value.

The procedures we employ here to elucidate key life history traits should also be applicable to the many thousands of species of other small Neotropical fishes for which reproductive life history biology is unknown. Much is to be gained from anchoring future phylogenetic studies of ecological distributions and phenotypic trait evolution in a firm understanding of reproductive life history. Moreover, a detailed understanding of reproductive life histories will form an essential baseline for monitoring the long-term effects of habitat degradation and climate change in the Neotropics.

## Supporting information

S1 Appendix Summary of sampling effort (n events per month and timed sampling effort in hours/month) for two whitewater floodplain lake sites (F1 and F2) and four terra firme stream sites (T1-T4). (PDF)

S2 Appendix Database of *Brachyhypopomus* from quantitative samples including species, year-group (for *B*. *beebei*), date and time of capture, site, habitat, physico-chemical water parameters, sex and gonad stage (see Table 1), and body size/mass, gonad mass, and liver mass. (XLSX)

S3 Appendix Equivalence of the gonad staging scheme in Table 1 to the schemes of Waddell and Crampton [49] and Núñez and Duponchelle [9]. (XLSX)

S4 Appendix Sagittal otolith annulus counts and accompanying specimen data for nine species of *Brachyhypopomus*. (XLSX)

S5 Appendix Data and measurements used for marginal increment analysis of the otoliths of *Brachyhypopomus bennetti*. (XLSX)

S6 Appendix Size-frequency distributions for four rarer species of *Brachyhypopomus*, during the breeding-season months, with Gaussian distributions fitted by finite mixture models: *B*. *benjamini*, *B*. *brevirostris*, *B*. *flavipomus*, *B*. *regani*. (PDF)

S7 Appendix Data and measurements for size-frequency distributions reported in S6 Appendix. (XLSX)

S8 Appendix Sagittal otolith preparations for four rarer species of *Brachyhypopomus*: *B*. *benjamini*, *B*. *brevirostris*, *B*. *flavipomus*, *B*. *regani*. (PDF)
