# Supplementary material for: Reproductive life-history strategies in a species-rich assemblage of Amazonian electric fishes
Source: PLoS One. 2019 Dec 5;14(12):e0226095. doi: 10.1371/journal.pone.0226095 (PMC6894849; doi:10.1371/journal.pone.0226095)
Supplement: S1 Appendix — (PDF) [file pone.0226095.s001.pdf]

S1 Appendix

Summary of sampling effort (n events per month and timed sampling effort in hours/month) for two whitewater floodplain lake sites (F1 and F2) and four terra firme stream sites (T1-T4). We undertook higher sampling efforts during the peak electric fish breeding periods from Nov-May to maximize the recovery of mature individuals. Months are Mar 2013 to Mar 2014. Sites are: F1 = Cocha Capite (04°51'18"S, 073°40'37"W, 97 m above mean sea level [AMSL]); F2 = unnamed lake (04°51'59"S, 073°40'60"W, 97 m above mean sea level [AMSL]); T1 = Quebrada Chupiari (04°53'59"S, 073°38'51"W, 127 m AMSL); T2 = Quebrada Sapuenillo (04°54'17"S, 073°36'46"W, 124 m AMSL): T3 = Quebrada Salome (04°54'32"S, 073°35'36"W, 118 m AMSL); T4 = Quebrada Parnayari (04°54'49"S, 073°39'43"W, 109 m AMSL).

	Sampling events						Total	Hours sampled						Total
	F1	F2	T1	T2	T3	T4		F1	F2	T1	T2	T3	T4	
January	2	3	8	9	2	2	26	10	9.8	33	41	10	9.7	113.9
February	2	2	6	2	1	1	14	9.2	13	36	15	5.6	6.7	84.5
March	3	2	5	3	3	2	18	15	9.1	27	14	13	13	90.7
April	4	4	6	5	6	2	27	15	18	36	36	19	13	136.1
May	1	1	3	1	3	2	11	8	8.7	15	7.6	12	12	62.8
June	1	1	3	2	1	2	10	6	5	15	15	5.7	12	58
July	1	1	2	2	1	2	9	5	5	6.9	12	5.3	7.8	41.8
August	2	1	4	2	4	3	16	11	4.9	20	7.6	19	9.6	71.6
September	1	2	3	1	4	2	13	5.5	11	15	4.4	23	6.4	64.4
October	2	1	3	4	2	1	13	12	6.2	14	11	8.7	4.9	56.3
November	2	1	4	1	3	2	13	8.2	4.8	22	5	12	8.6	60.8
December	2	2	10	4	2	3	23	6.4	7.5	39	21	9.9	13	96.1
Total	23	21	57	36	32	24	193	111	102	277	189	142	116	**937**
Mean per month	1.9	1.8	4.8	3.0	2.7	2.0		9.3	8.5	23.1	15.8	11.8	9.6	
