# Supplementary material for: Reproductive life-history strategies in a species-rich assemblage of Amazonian electric fishes
Source: PLoS One. 2019 Dec 5;14(12):e0226095. doi: 10.1371/journal.pone.0226095 (PMC6894849; doi:10.1371/journal.pone.0226095)
Supplement: S6 Appendix — (PDF) [file pone.0226095.s006.pdf]

S6 Appendix

Size-frequency distributions for four species of *Brachyhypopomus* during the breeding-season months (terra firme stream: October-March; floodplain December-February). Data for *B. benjamini* are from terra firme streams during the main study period (2013-2014). Data for *B. brevirostris*, *B. flavipomus*, and *B. regani* are from non-quantitative samples taken in 2015 from nearby floodplain lakes of the Ucayali River floodplain in the Pacaya Samiria Reserve. The ordinate in these plots refers to the proportional abundance of individuals belonging to each histogram bin. Note the unimodal distribution of sizes in the putative annual semelparous species *B. benjamini*, *B. flavipomus*, and *B. regani*. *Brachyhypopomus brevirostris* shows two size groups containing sexually mature adults which we assume represent 0+ and 1+ year-groups in a two-year iteroparous life history. We also recovered a third smaller group comprising only immature individuals. Because *B. brevirostris* is known to attain sexual maturity in its first year (Kirschbaum & Schugardt 2002) we presume that the smaller group corresponds to a newly recruited group of juveniles within the 0+ group. The cut-off between the putative 0+ and 1+ year-groups measured by the intersection of the two Gaussian distributions is at 150 mm LEA, see vertical dotted line. See S7 Appendix for supporting data.

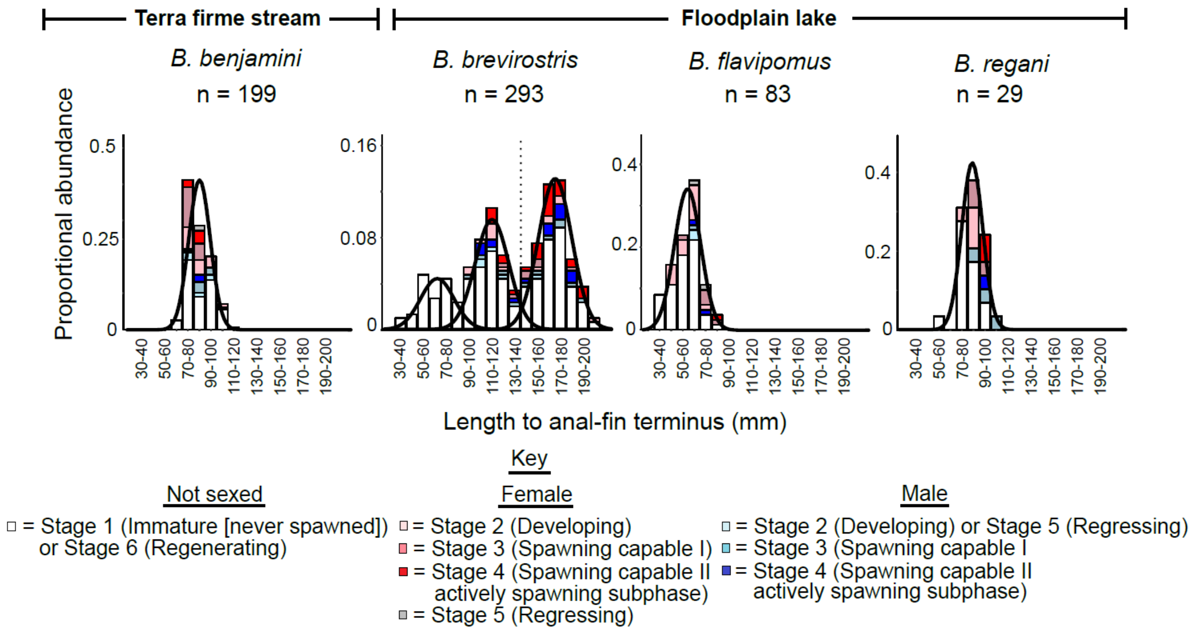

References:

Kirschbaum F, Schugardt C. Reproductive strategies and developmental aspects in mormyrid and gymnotiform fishes. Journal of Physiology-Paris. 2002;96:557-66.
