# Supplementary material for: Reproductive life-history strategies in a species-rich assemblage of Amazonian electric fishes
Source: PLoS One. 2019 Dec 5;14(12):e0226095. doi: 10.1371/journal.pone.0226095 (PMC6894849; doi:10.1371/journal.pone.0226095)

S8 Appendix

Sagittal otolith preparations for representative stage 4 individuals of four rarer species of *Brachyhypopomus*. Dotted lines are superimposed to highlight the positions of the otolith core and dark annuli. Note the single dark annulus in *B. benjamini*, *B. flavipomus*, and *B. regani*. Note the single dark annulus in a putative 0+ year group of *B. brevirostris*, but two dark annuli in a putative 1+ year group.

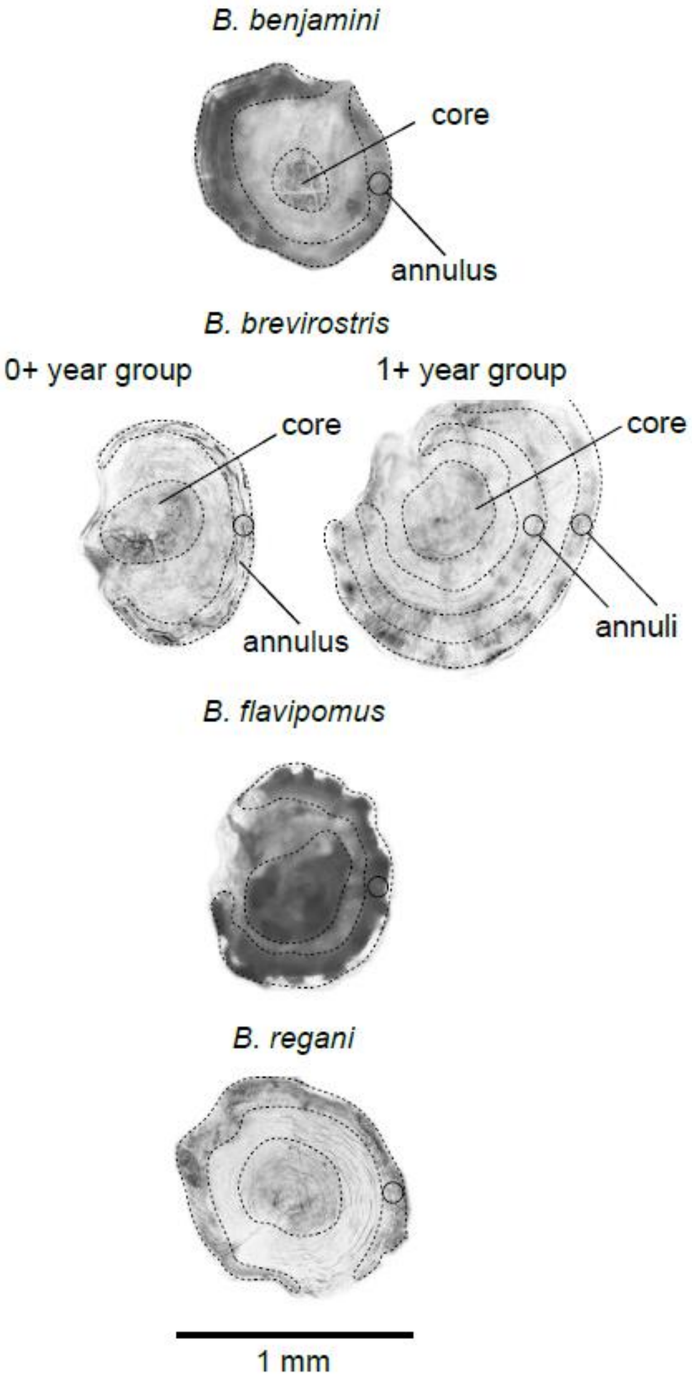

Supplement: S8 Appendix — (PDF) [file pone.0226095.s008.pdf]
